# Single cell eQTL analysis identifies cell type-specific genetic control of gene expression in fibroblasts and reprogrammed induced pluripotent stem cells

**DOI:** 10.1186/s13059-021-02293-3

**Published:** 2021-03-05

**Authors:** Drew Neavin, Quan Nguyen, Maciej S. Daniszewski, Helena H. Liang, Han Sheng Chiu, Yong Kiat Wee, Anne Senabouth, Samuel W. Lukowski, Duncan E. Crombie, Grace E. Lidgerwood, Damián Hernández, James C. Vickers, Anthony L. Cook, Nathan J. Palpant, Alice Pébay, Alex W. Hewitt, Joseph E. Powell

**Affiliations:** 1 Garvan-Weizmann Centre for Cellular Genomics, Garvan Institute of Medical Research, Darlinghurst, Sydney, Australia; 2 Institute for Molecular Bioscience, University of Queensland, Brisbane, Australia; 3 Centre for Eye Research Australia, Royal Victorian Eye and Ear Hospital, Melbourne, Australia; 4 Department of Surgery, The University of Melbourne, Melbourne, Australia; 5 Department of Anatomy and Physiology, The University of Melbourne, Melbourne, Australia; 6 Wicking Dementia Research and Education Centre, University of Tasmania, Hobart, Australia; 7 School of Medicine, Menzies Institute for Medical Research, University of Tasmania, Hobart, Australia; 8 UNSW Cellular Genomics Futures Institute, School of Medical Sciences, University of New South Wales, Sydney, Australia

**Keywords:** Expression quantitative trait loci (eQTLs), Single cell RNA-sequencing (scRNA-seq), Induced pluripotent stem cells (iPSCs)

## Abstract

**Background:**

The discovery that somatic cells can be reprogrammed to induced pluripotent stem cells (iPSCs) has provided a foundation for in vitro human disease modelling, drug development and population genetics studies. Gene expression plays a critical role in complex disease risk and therapeutic response. However, while the genetic background of reprogrammed cell lines has been shown to strongly influence gene expression, the effect has not been evaluated at the level of individual cells which would provide significant resolution. By integrating single cell RNA-sequencing (scRNA-seq) and population genetics, we apply a framework in which to evaluate cell type-specific effects of genetic variation on gene expression.

**Results:**

Here, we perform scRNA-seq on 64,018 fibroblasts from 79 donors and map expression quantitative trait loci (eQTLs) at the level of individual cell types. We demonstrate that the majority of eQTLs detected in fibroblasts are specific to an individual cell subtype. To address if the allelic effects on gene expression are maintained following cell reprogramming, we generate scRNA-seq data in 19,967 iPSCs from 31 reprogramed donor lines. We again identify highly cell type-specific eQTLs in iPSCs and show that the eQTLs in fibroblasts almost entirely disappear during reprogramming.

**Conclusions:**

This work provides an atlas of how genetic variation influences gene expression across cell subtypes and provides evidence for patterns of genetic architecture that lead to cell type-specific eQTL effects.

**Supplementary Information:**

The online version contains supplementary material available at 10.1186/s13059-021-02293-3.

## Background

Mapping expression quantitative trait loci (eQTLs) is a powerful method to study how common genetic variation between individuals influences gene expression [1, 2]. To date, nearly all eQTL studies have been conducted while interrogating ‘bulk’ samples, where the RNA is collected from millions of lysed cells, and therefore, gene expression represents an average across all cells in a sample. However, even ‘bulk’ eQTL studies in different tissues [3, 4] and cultured cell lines [5, 6] have revealed specificity in both the presence and allelic effects of eQTLs [7, 8]. Single cell approaches have already revealed that stem cell cultures do not contain a single homogeneous cell type [5, 6, 9], but instead consist of multiple cell types that have unique transcriptional profiles. For this study, we harnessed recent technological advances for high-throughput generation of single cell data that leveraged cell multiplexing from multiple donors [10–12]. This experimental framework enabled the identification of cell type-specific genetic effects on gene expression which revealed eQTLs that were cell type specific and that would not be detected by ‘bulk’ approaches.

Previous studies have identified cell type-specific eQTLs using scRNA-seq which were unobservable in bulk RNA-sequence studies [13–17]. The first study to report this enhanced cell type-specific eQTL detection from scRNA-seq investigated 92 genes measured in 1440 single cells from lymphoblastoid cell lines in 15 individuals [15]. In the current study, we set out to understand the impact of common genetic variants on gene expression in fibroblast and reprogrammed iPSC cell types through eQTL mapping at the level of cell subpopulations.

## Results

**Fig. 1:**
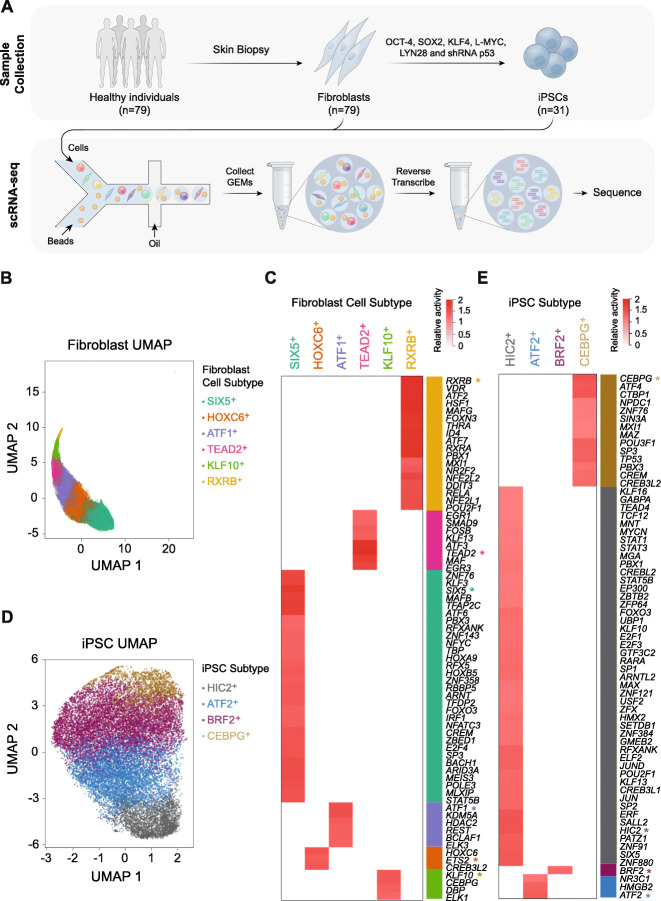
Fibroblast and iPSC cluster characterisation. **a** This study used skin biopsies to generate fibroblasts from 79 healthy volunteers and reprogrammed them into induced pluripotent stem cell (iPSC) lines for 31 of the original 79 individuals. **b** Six fibroblast subtypes were identified from the transcriptional profiles of 64,018 single fibroblast cells. **c** The 50 TFs with the highest relative activities as identified by SCENIC in the six fibroblast subtypes. **d** Four iPSC subtypes were identified from 19,967 single iPSCs. **e** The 50 TFs with the highest relative activities as identified by SCENIC in the four iPSC subtypes. Asterisk indicates the genes used to name each subtype

To identify cell type-specific eQTLs in an unbiased manner, we generated scRNA-seq expression profiles of 83,985 cells—64,018 cultured dermal fibroblasts, generated from skin biopsies from 79 unrelated individuals, and 19,967 iPSCs reprogrammed from 31 of the dermal fibroblast lines (Fig. 1a). After quality control, we used an unsupervised clustering approach [18] to identify six types of fibroblasts and four types of iPSCs (Fig. 1b, c). Fibroblast and iPSC types contained equal distributions of individual donors, pool batches and cell cycle states (Additional file 1: Figure S1 and S2). Cell types were classified based on the relative activity of the regulating transcription factors in fibroblasts (SIX5^+^, HOXC6^+^, ATF1^+^, TEAD2^+^, KLF10^+^ and RXRB^+^) and iPSCs (HIC2^+^, ATF2^+^, BRF2^+^ and CEBPG^+^) (Fig. 1d, e; Additional file 2: Table S1 and Additional file 3: Table S2; and Table 1). Further, pseudo-trajectory analysis demonstrated that the identified cell types were positioned along a clear lineage trajectory for both fibroblast and iPSC types which was exemplified by the top differentially expressed genes (Additional file 1: Figure S3–4, Additional file 4: Table S3 and Additional file 5: Table S4). We also used an unbiased approach to classify cells against reference transcriptome profiles from the human primary cell atlas [19, 20], which demonstrated that the majority of fibroblasts mapped to the fibroblast or mesenchymal stem cell (MSC) reference, while the majority of iPSCs mapped to the iPSC or embryonic stem cell references (Additional file 1: Figure S5A-B). Due to the phenotypic and transcriptional similarities of fibroblasts and MSCs (Additional file 1: Figure S5C), it is not surprising that some fibroblast cells mapped to the MSC reference [21].

**Table 1:** Summary of fibroblast type *cis-*eQTL. The median number of cells per individual, the number of significant eSNPs detected, the number of significant eGenes detected and the number of unique eGenes per cell type are enumerated

Cell type	Median cells per individual	Number of significant eSNP-eGene pairs	Number of significant eGenes	Unique genes with cis-eQTL
Fibroblast SIX5+	282	13,886	664	383
Fibroblast HOXC6+	253	23,348	1030	615
Fibroblast ATF1+	175	20,105	916	500
Fibroblast TEAD2+	46	6351	402	232
Fibroblast KLF10+	36	4567	299	163
Fibroblast RXRB+	7	2883	517	339
iPSC HIC2+	240	52	6	5
iPSC ATF2+	176	14	3	2
iPSC BRF2+	74	52	11	7
iPSC CEBPG+	41	695	68	55

**Fig. 2:**
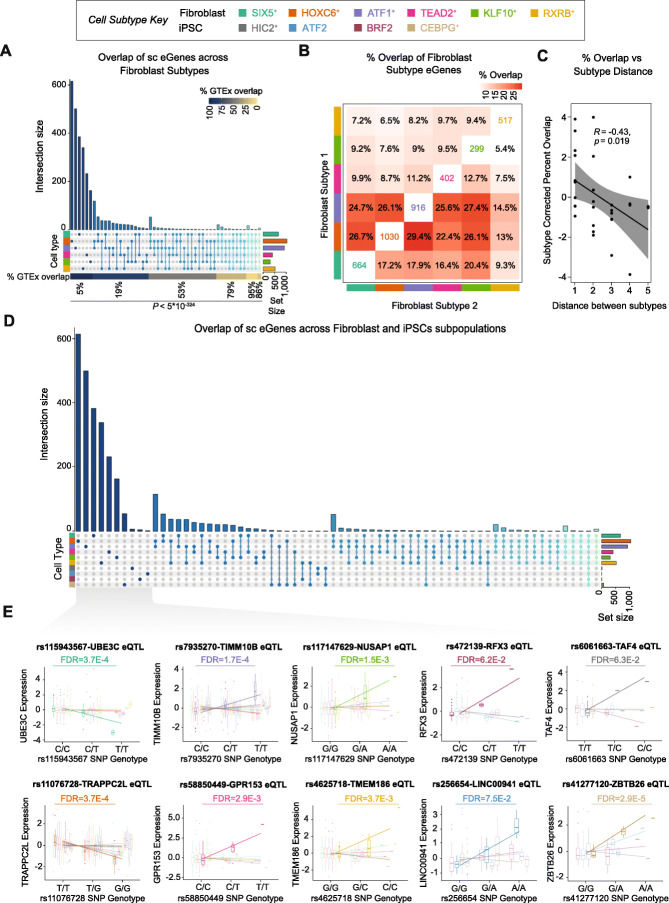
Identification of single cell eQTLs in fibroblast and iPSC subtypes. **a** The majority of single cell (sc) eGenes in fibroblasts are subtype specific. Further, the single cell eGenes that were detected in two or more fibroblast subtypes were significantly more likely to be detected as eQTLs in bulk fibroblast RNA-sequence data from the gene tissue expression (GTEx) database (*p* < 5 × 10^−324^, Cochran-Armitage test). **b** The total number of eGenes (on the diagonal) and percent that are also observed in other fibroblast subtypes further shows that most eGenes are unique to a given subtype. **c** Neighbouring fibroblast cell types were more likely to share common eGenes than distant cell types. **d** iPSC types demonstrate a similar level of cell specificity and none of the single cell eGenes-eSNP pairs that were observed in fibroblasts were observed in the iPSC subtypes that were generated from the same individuals. **e** Top eQTLs unique to a specific cell type exemplify the cell type specificity

We subsequently tested for *cis*-eQTLs independently in each of the 10 cell types. We identified a total of 46,103 eQTLs for 2958 genes (FDR < 0.1) across all cell types—45,503 eQTLs for 2887 genes in fibroblast cell types and 810 *cis*-eQTLs for 86 genes in iPSC cell types (Additional file 7: Table S6 and Additional file 8: Table S7, Table 1). The majority of *cis*-eQTLs were predominantly cell type specific, with 77.6% of the eGenes—genes that have an eQTL—(71.5% of the *cis*-eQTLs) identified in only one fibroblast type (Fig. 2a, b and Additional file 1: Figure S6A). However, neighbouring fibroblast types were more likely to share common eGenes than distant fibroblast types (Fig. 2b, c). iPSC types also demonstrated a high percentage of cell type-specific eQTLs with 97.2% of the eGenes (99.6% of the *cis*-eQTLs) identified in only one iPSC type (Additional file 1: Figure S6B-C). Since each cell type was characterised by activity of a specific set of transcription factors (Fig. 1c, e), we tested whether any of the eQTLs were predicted to alter transcription factor binding in those cell types and identified multiple loci that were predicted to alter key transcription factor binding (Additional file 8: Table S7).

Cell type ubiquitous eQTLs (shared across all fibroblasts or iPSC cell types) were rare, with seven eGenes in fibroblasts (Fig. 2a, Additional file 1: Figure S6A and S7) and none in iPSCs (Additional file 1: Figure S6B). Looking across the cell reprogramming event, we observed a complete lack of shared eQTLs between fibroblasts and iPSCs. Only 14 genes had eQTLs in both fibroblasts and iPSCs (Fig. 2d), but none of those shared a common eSNP—a SNP significantly associated with an eGene—or eSNPs in linkage disequilibrium with one another (*r*^*2*^ < 0.2), which indicates that their expression is likely associated with independent loci (Fig. 2d and Additional file 1: Figure S8). These cell type-specific eQTLs are clearly exemplified by top eQTLs identified for each of the 10 cell types (Fig. 2e).

We then investigated whether the eQTLs identified in fibroblasts replicated in bulk RNA-sequence data from the Genotype-Tissue Expression database (GTEx, cultured fibroblasts *n* = 483) [22]. Only 41.1% of the 45,503 eQTLs identified in the six fibroblast types replicated in GTEx, although they demonstrated a consistent shared direction of allelic effects. One explanation for this observation is that bulk RNA-seq approaches mask cell type-specific effects through averaged gene expression across cells. Therefore, we hypothesised that cell type ubiquitous eQTLs (from the single cell analysis) would have higher replication rates compared to cell type-specific eQTLs. eQTLs that were shared across multiple scRNA-seq fibroblast cell types showed a highly significant difference compared with eQTLs that were significant in just one fibroblast type (*p* < 5 × 10^−324^ for eGenes and *p* = 6× 10^−150^ for eSNPs; Fig. 2a and Additional file 1: Figure S6A). Further, we identified that the allelic effect size of the eGenes and eQTLs in GTEx cultured fibroblasts was positively correlated with the number of fibroblast types where those eGenes and eQTL were significant (Additional file 1: Figure S9). These results indicate that eQTL mapping using bulk RNA-sequence data is likely not sensitive enough to identify fibroblast type-specific eQTLs.

Based on our initial observation that cell type eQTL effects are highly specific, we next sought to identify how different types of genetic architecture and gene expression patterns contributed to cell type-specific effects in fibroblasts and iPSCs.

One potential explanation for the cell type-specific eQTL detection is that the gene is only expressed in one cell type, and therefore, we would not expect to observe an eQTL in a cell type that does not express the gene. To evaluate this possibility, we correlated the expression of each eGene, with its expression levels in each of the other cell types (Additional file 1: Figure S10). Gene expression was highly correlated, which indicates that cell type-specific eQTL is not a function of cell type-specific gene expression. Another possible explanation for the cell type-specific eQTLs is low statistical power to detect eQTLs in multiple cell types. We tested this hypothesis in two ways: (1) we correlated the eQTL effect sizes between different cell types and (2) we implemented an empirical framework to test for enrichment of cell type-specific eGenes in other cell types. The cell type-specific eQTL allelic effects (betas) were not well correlated in other cell types unless they were already identified as significant in the other cell type (Additional file 1: Figure S11). However, there was some enrichment of the test statistic across cell types—mostly for fibroblast types that were similar to one another such as SIX5^+^ and HOXC6^+^ (Additional file 1: Figure S12). While larger studies will be required to fully elucidate the full degree of cell type specificity, these results suggest that many of the eQTLs identified are cell type specific. Therefore, we conclude that the majority of cell type-specific eQTLs that we have identified were not a result of differences in gene expression or due to lack of statistical power. We next set out to interrogate eGenes that were in common between multiple cell types.

**Fig. 3:**
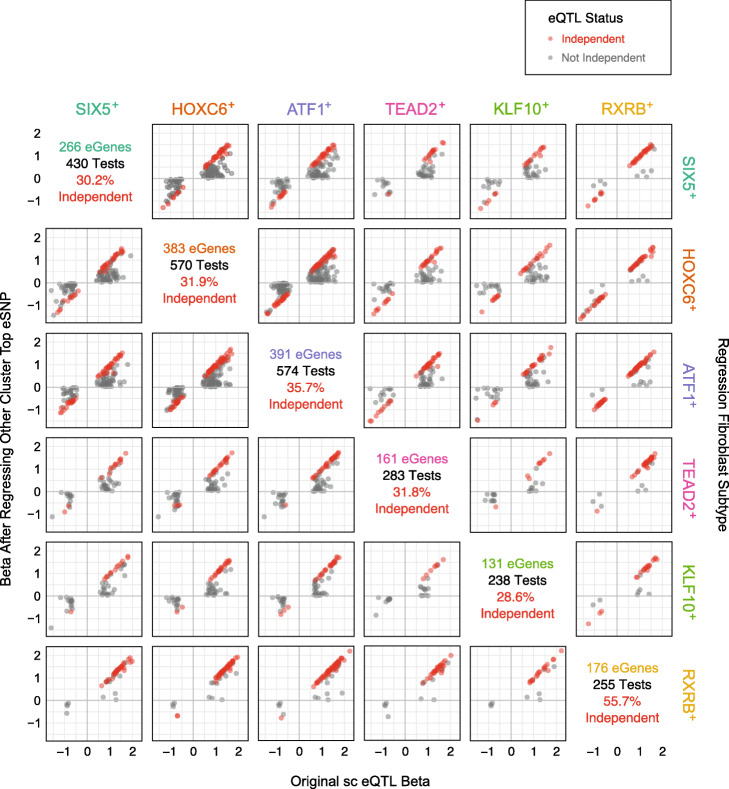
eGene comparison across fibroblast subtypes. eGenes that were shared between at least two fibroblast subtypes were tested for independence. The top eSNP for eGenes that were shared between two fibroblast subtypes was regressed from the other subtype in order to test if those were independent eSNP loci. Many (40–73%) of the fibroblast top eSNPs remained significant after regression of the top eSNP from another fibroblast subtype

We identified 283 eGenes that were significant in multiple cell types, but which had different top eSNPs—255 eGenes in at least two fibroblast types, no eGenes with different top eSNPs in iPSC types and 11 eGenes with different top eSNPs in a fibroblast type and an iPSC type. In these instances, we considered two alternative hypotheses: (1) that there was one eQTL shared between cell types but that it was tagged by a different top eSNP in each cell type, or (2) that there were two independent cell type-specific eQTLs for the same gene. To address these hypotheses, we tested whether the top eSNP in a given cell type was still significantly associated with gene expression after correcting for the top eSNP in the other cell type—the same method used for conditional eQTL analyses [23]. A significant association of the SNP with the eGene expression after correction for the other eSNP would indicate that the two eSNPs were not tagging the same eQTL and were, therefore, independent loci. The analysis identified that between 28.6 and 55.7% of these loci for a given fibroblast type were independent (Fig. 3 and Additional file 9: Table S8), and 100% of the eGenes shared between the fibroblast and iPSC types were also independent loci (Additional file 9: Table S8). These results denote that many of the eGenes that were shared between multiple cell types are in fact regulated by different loci, providing further support to our previous finding that the majority of eQTLs are cell type specific.

**Fig. 4:**
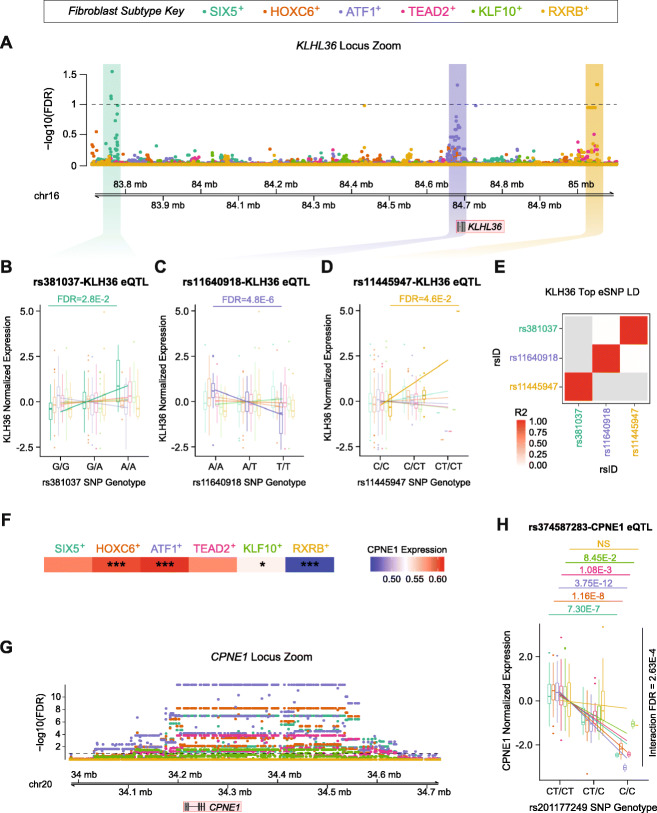
Examples of eQTLs identified in fibroblast and iPSC subtypes. **a** The *KLHL36* gene was significant in three different fibroblast subtypes but with different top eSNPs for each cell type. **b** The top SNP for the SIX5^+^ fibroblast cell type was rs381037 and demonstrated a significant association with KLHL36 expression in just the SIX5^+^ fibroblast cell type. **c** rs11604918 was a significant SNP in just the ATF1+ fibroblast cell type. **d** The rs11445947 SNP was the most significant eSNP for KLHL36 expression in the RXRB^+^ cell type and did not demonstrate a significant association in any other cell type. **e** The three top eSNPs associated with KLHL36 expression were not in linkage disequilibrium. **f** CPNE1 was differentially expressed in HOXC6^+^, ATF1^+^, KLF10^+^ and RXRB^+^ fibroblast cell types. **g** CPNE1 was a significant eGene in five of the six fibroblast subtypes. **h** Further, the rs3474587283-CPNE1 eQTL demonstrated striking subtype by SNP interaction. *p < 0.05; **p < 0.01; ***p < 0.001; NS non-significant

Indeed, we identified that even though the Kelch Like Family Member 36 (*KLHL36*) gene was a significant eGene in three fibroblast types, it was regulated by independent loci in each cell type (Fig. 4a–d). Further, the top eSNPs for each locus were not in LD with one another (*r*^*2*^ < 0.1) (Fig. 4e). *KLHL36* is highly expressed in fibroblasts compared to other cell types in the Genotype-Tissue Expression (GTEx) database (Additional file 1: Figure S13) suggesting that it may play an important role in fibroblast biology. Further, *KLHL36* is part of the E3 ubiquitin ligase family which has been implicated in skin fragility [24] and fibroblast pseudopodia function [25]—again highlighting the potential role of this gene in fibroblast biology and physiology.

Next, we investigated the 153 eGenes that shared at least one significant eSNP-eGene pair across multiple cell types. We evaluated the potential interactions between cell type and eSNP that would lead to a difference in magnitude of the allelic effect in different cell types. In cases where multiple eSNP-eGene pairs were significant for the same eGene across multiple cell types, we tested the eSNP-eGene pair with the largest beta difference between two fibroblast types. This analysis identified 42 (21.2%) significant eSNP-fibroblast type interactions at an FDR of 0.05 (Additional file 10: Table S9). We identified a number of ubiquitous eQTLs, whose allelic effects were significantly different across cell types.

Next, we asked whether any of the eQTLs that we identified might impact fibroblast reprogramming to iPSCs. We first asked whether there were eQTLs for any genes that characterise cell types as they transition from fibroblasts to iPSCs. We identified multiple eQTLs for genes that characterised cell types during the transition from fibroblasts to iPSCs (Additional file 1: Figure S14A). In addition, 76.5% of those reprogramming eQTLs were unique to one fibroblast subtype which suggests that these genetic variants may impact reprogramming efficiency to varying degrees depending on the cell type. Further, we tested for eQTLs in regions differentially methylated post reprogramming (Additional file 1: Figure S14B) and in regions that are incompletely reprogrammed in some iPSCs relative to embryonic stem cells (ESCs) (Additional file 1: Figure S14C). In both cases, we identified multiple eQTLs, the majority of which were cell type specific—68.6% in regions differentially methylated between fibroblasts and iPSCs and 62.1% in regions differentially methylated between iPSCs and ESCs, indicating that these genetic variants may influence iPSC reprogramming. Of interest, this analysis identified genes that have previously been associated with reprogramming efficiency such as *DNAJ15* [26] or previously been reported as eQTLs such as *MICA* [27] and *CPNE1* [28].

In addition, *CPNE1* was differentially expressed between cell types in our dataset (Additional file 1: Figure S4F and Additional file 4: Table S3) and the rs374587283-*CPNE1 cis*-eQTL demonstrated a significant SNP-cell type interaction (*p* = 2.64.9 × 10^−04^; Fig. 4g, h). CPNE1 is a calcium-dependent binding protein that is thought to play an important role in membrane trafficking, and is important for neuronal cell differentiation [29, 30]. Therefore, this eQTL could impact the differentiation potential of iPSC lines toward neuronal lineages.

## Discussion

We set out to identify and define the dynamics of eQTLs in fibroblasts and fibroblast-derived iPSC types. Collectively, our results provide evidence that there is a high degree of cell type-specific gene regulation that is not captured with bulk RNA-seq. Our results indicate that even when the same eGene is observed in different cell types, the allelic effect may be altered in different cell types or may be regulated by different loci entirely. Our findings support previous reports that many cell type-specific eQTLs are not detected using bulk RNA-sequencing and that scRNA-seq can be utilised to enhance eQTL detection [31].

These results contrast slightly with GTEx [22] that showed that multiple eQTLs are common across tissues. However, it is important to note that in GTEx, the RNA expression levels are the averaged signal from all cell types in the tissue biopsy. While the cellular composition of tissues differs, most tissues share common cell types such as epithelial cells, circulating immune cells and adipocytes. Therefore, under a scenario of highly cell type-specific eQTLs, we would expect to see shared eQTLs between tissues due to the common cell types that are shared between multiple tissues. Likewise, truly cell type ubiquitous eQTLs would replicate at a higher frequency between single cell eQTLs and GTEx—as we observe here (Fig. 2). Nevertheless, the results we present here strongly support the hypothesis of true cell type-specific eQTLs as we observe little correlation of the allelic effects (betas) between cell types. However, to adequately uncover the relationship between bulk and single cell eQTLs, it is likely that future studies which generate both types of data will be required.

In addition, our analyses identified novel *cis*-eQTLs for genes that characterise cell types during iPSC reprogramming [32], located in functional genomic regions that reflect reprogramming efficiency based on epigenetic profiles [33, 34]. Those loci also demonstrated important cell type specificity that could impact iPSC reprogramming and pluripotency in a cell type-specific manner. However, additional studies will be required to fully understand the mechanisms behind how these genetic loci might impact iPSC reprogramming in lines that are derived from different individuals. Specifically, CellTag [35, 36] or other barcoding methods will enable cells to be both collected and tracked throughout the entire reprogramming event or differentiation lineage.

scRNA-seq provides a number of advantages over bulk RNA-sequencing for eQTL mapping. Specifically, scRNA-seq enables cell types to be identified in an unbiased manner before eQTL detection. Therefore, even cell types that have previously not been described or well characterised can be identified and separated for eQTL mapping, thereby decreasing the measurement noise that is introduced due to heterogeneity of cells in bulk RNA-sequence profiling. Furthermore, scRNA-seq enables the cells from multiple individuals to be pooled in a single experiment, thereby decreasing technical batch effects that can confound biological variation between individuals.

Larger studies will be required to fully parse out the cell type-specific effects.

## Conclusions

This study has provided a map of eQTLs in fibroblast and fibroblast-derived iPSC types that will be an important reference for future studies in iPSC-derived cell types.

## Methods

### Participant recruitment and ethics approval

Experimental work was approved by the Human Research Ethics committees of the Royal Victorian Eye and Ear Hospital (11/1031), University of Melbourne (1545394) and University of Tasmania (H0014124) in accordance with the requirements of the National Health & Medical Research Council of Australia (NHMRC) and conformed with the Declaration of Helsinki [37].

### Fibroblast culture

Human skin punch biopsies were obtained from subjects over the age of 18 years. Fibroblasts were cultured in DMEM high glucose supplemented with 10% foetal bovine serum (FBS), L-glutamine, penicillin (100 U/mL) and streptomycin 100 (μg/mL) (all from Thermo Fisher Scientific, USA). All cell lines were mycoplasma-free (MycoAlert mycoplasma detection kit, Lonza, Switzerland).

### Generation and maintenance of iPSCs

Human iPSCs were reprogrammed from fibroblast cultures by nucleofection (Amaxa™ Nucleofector™) of episomal vectors expressing *OCT-4*, *SOX2*, *KLF4*, *L-MYC*, *LIN28* and shRNA against p53 [38], in feeder- and serum-free conditions using TeSR™-E7™ medium (STEMCELL Technologies, Canada) and selected by sorting with anti-human TRA-1-60 Microbeads using a MultiMACS (Miltenyi, Germany) as described in [39] and [40]. Cells were maintained on vitronectin XF™ (STEMCELL Technologies)-coated plates using TeSR™-E8™ (Stem Cell Technologies). At passage eight, cells were assessed for quality control as described previously [40].

### iPSC quality control

Pluripotency was assessed by immunochemistry for expression of OCT3/4 (sc-5279, Santa Cruz Biotechnology, USA) and TRA-1-60 (MA1-023-PE, Thermo Fisher Scientific). Copy number variation (CNV) analysis of original fibroblasts and iPSCs was performed using Illumina HumanCore Beadchip arrays with PennCNV [41, 42] and QuantiSNP [42] with default parameter settings. Chromosomal aberrations were deemed to involve ≥ 20 contiguous SNPs or a genomic region spanning ≥ 1 MB [41, 42]. The B allele frequency (BAF) and the log R ratio (LRR) were extracted from GenomeStudio (Illumina, USA) for representation.

### Generating the single cell RNA-sequence data

Viable cells were sorted on a BD Influx cell sorter (Becton-Dickinson) using propidium iodide into Dulbecco’s phosphate-buffered saline (PBS) + 0.1% bovine serum albumin and retained on ice. Sorted cells were counted and assessed for viability with Trypan Blue using a Countess automated counter (Invitrogen) and then resuspended at a concentration of 800–1000 cells/μL (8 × 10^5^ to 1 × 10^6^ cells/mL). Final cell viability estimates ranged between 92 and 96%.

Single cell suspensions were loaded onto 10X Genomics Single Cell 3′ Chips along with the reverse transcription (RT) mastermix as per the manufacturer’s protocol for the Chromium Single Cell 3′ Library (10X Genomics; PN-120233), to generate single cell gel beads in emulsion (GEMs). Reverse transcription was performed using a C1000 Touch Thermal Cycler with a Deep Well Reaction Module (Bio-Rad) as follows: 55 °C for 2 h, 85 °C for 5 min, hold 4 °C. cDNA was recovered and purified with DynaBeads MyOne Silane Beads (Thermo Fisher Scientific; Cat# 37002D) and SPRIselect beads (Beckman Coulter; Cat# B23318). Purified cDNA was amplified as follows: 98 °C for 3 min; 12x (98 °C for 15 s, 67 °C for 20 s, 72 °C for 60 s); 72 °C for 60 s; hold 4 °C. Amplified cDNA was purified using SPRIselect beads and sheared to approximately 200 bp with a Covaris S2 instrument (Covaris) using the manufacturer’s recommended parameters. Sequencing libraries were generated with unique sample indices (SI) for each chromium reaction. Libraries were multiplexed and sequenced on an Illumina NextSeq 500 (NextSeq control software v2.0.2/Real Time Analysis v2.4.11) using a 150-cycle NextSeq 500/550 High Output Reagent Kit v2 (Illumina, FC-404-2002) in standalone mode as follows: 98 bp (Read 1), 14 bp (I7 Index), 8 bp (I5 Index) and 10 bp (Read 2).

### scRNA-seq Cellranger processing

Processing of the sequencing data into transcript count tables was performed using the Cell Ranger Single Cell Software Suite by 10X Genomics [43]. Raw base call files from the NextSeq 500 sequencer were demultiplexed, using the cellranger mkfastq pipeline, into sample-specific FASTQ files. These FASTQ files were then processed with the cellranger count pipeline where each sample was processed independently. First, cellranger count used STAR to align cDNA reads to the hg19 human reference transcriptome, which accompanied the Cell Ranger Single Cell Software Suite [44]. We note that, since the expression data is limited to the 3′ end of a gene and we used gene-level annotations, differences between reference versions, such as GRCh38, are unlikely to significantly alter conclusions. Aligned reads were filtered for valid cell barcodes and unique molecular identifiers (UMIs) and observed cell barcodes were retained if they were 1-Hamming-distance away from an entry in a whitelist of known barcodes. UMIs were retained if they were not homopolymers and had a quality score > 10 (90% base accuracy). Cellranger count corrected mismatched barcodes if the base mismatch was due to sequencing error, determined by the quality of the mismatched base pair and the overall distribution of barcode counts. A UMI was corrected to another, more prolific UMI if it was 1-Hamming-distance away and it shared the same cell barcode and gene. Cellranger count examined the distribution of UMI counts for each unique cell barcode in the sample and selected cell barcodes with UMI counts that fell within the 99th percentile of the range defined by the estimated cell count value. The default estimated cell count value of 3000 was used for this experiment. Counts that fell within an order of magnitude of the 99th percentile were also retained. The resulting analysis files for each sample were then aggregated using the cellranger aggr pipeline, which performed a between-sample normalisation step and merged all samples into one. Post-aggregation, the count data was processed and analysed using a comprehensive pipeline assembled and optimised in-house as described below.

To pre-process the mapped data, we constructed a cell quality matrix based on the following data types: library size (total mapped reads), the total number of genes detected, percent of reads mapped to mitochondrial genes and percent of reads mapped to ribosomal genes (Additional file 1: Figure S15). Cells that had any of the four parameter measurements that were greater than 3x median absolute deviation (MAD) of all cells were considered outliers and removed from subsequent analysis. In addition, we applied two thresholds to remove cells with mitochondrial reads above 20% or ribosomal reads above 50%. To exclude genes that were potentially detected from random noise, we removed genes that were detected in fewer than 1% of all cells. These quality control filters resulted in consistent total reads per individual and per pool in both fibroblasts and iPSCs (Additional file 1: Figure S16). Before normalisation, abundantly expressed ribosomal genes and mitochondrial genes were discarded to minimise the influence of those genes in driving clustering and differential expression analysis.

### Demultiplexing

We adapted the Demuxlet method to our 10x scRNA-seq data [16]. The likelihood that a cell originated from a sample is the cumulative likelihood of single nucleotide polymorphism genotypes identified in each cell. We calculated posterior probability of a genotype *g* identified for a cell based on scRNA-seq data given the DNA data from the imputed BeadChip genotypes. Since the single cell SNP genotype data is sparse, to increase the coverage of SNPs called from scRNA-seq data that are in the SNP genotype data, we imputed SNP genotypes using the haplotype reference panel. We applied an ensemble approach using the outputs from pre-imputed genotype data, imputed genotype likelihood data, and impute genotype dosage data, increased the singlet probabilities from Demuxlet (Additional file 1: Figure S17). The ensemble approach enabled the unique donor assignment of 90.6% of all cells, with high confidence to each sample, where Demuxlet predicted no ambiguously assigned droplets. Of note, 100% of the cells before Demuxlet were identified in the cellranger pipeline as a singlet. Demuxlet identified 90.6% of all cellranger singlet cells as ‘real’ single cells. Therefore, these cells were ascertained as singlets. To recover the cell assignment to the remaining 9.4% cellranger singlets, predicted as doublets by Demuxlet, we utilised gene expression matrix to model cell doublets, using a simulation-based approach [45]. For each cell that was identified as a singlet by both Demuxlet and the doublet expression simulation, it was assigned to a donor based on the highest likelihood probability from Demuxlet. Doublets identified by Demuxlet or the doublet expression simulation were removed before downstream processing and analysis.

### Normalisation

Normalisation was conducted at four levels: between samples within a pool, between pools, between cells and between clusters. The between-pool normalisation followed the subsampling strategy in the cellranger pipeline, where the reads, genes and cells were randomly subsampled following subsampling rates determined by the total read per sample and binomial distribution [46]. Four pools were randomly multiplexed into one sequencing lane. For cell-to-cell normalisation, a cell-pooling strategy was applied to circumvent the zero-inflation issue, as described by Lun et al. [47]. Between-pool normalisation followed Combat parametric empirical Bayesian strategy. To select the normalisation strategy, we compared results from using Combat, RUV and SCRAN methods by using *k*-BET batch-effect scores [18]. We found that a combination of SCRAN normalisation followed by Combat was superior in reducing batch effects compared to other methods, consistent with the results reported by Buttner and colleagues [18]. Prior to eQTL analysis, the mean expression of each gene per individual per cell subpopulation was computed, quantile normalised and z-transformed for eQTL mapping.

### Imputation and quality control of genotype data

The 79 cell lines were genotyped by Infinium HumanCore-24 v1.1 BeadChip assay (Illumina). GenomeStudioTM V2.0 (Illumina) was used for SNP genotype calling of the BeadChip data (total 306,670 SNPs for one assay). The full genotype report files were reformatted into Plink map, fam and lgen files and were then converted into variant calling format (vcf) using custom shell scripts and Plink2 [48]. Plink2-converted files contained predicted reference and alternative alleles with no information for homozygous genotypes, which were fixed using the GenomeStudio report file and a custom script. For each sorted, indexed vcf file (separated by chromosomes), a strand fixing step was performed using bcf fixref function [49]. Prior to imputation, Eagle V.2.3.5 was used for haplotype phasing the strand-fixed genotype vcf files [50]. The phased data were imputed based on the 1000 genome phase 3 reference panel (2535 samples) using the minimac3 program in the Michigan Imputation server [51].

### Cell type classification and annotation

We combined all cells from the fibroblasts and iPSC pools separately. We normalised and clustered the cells in these two datasets to ensure that the clustering was not affected by pool-specific data processing. We performed clustering using the SCORE method to identify subpopulations of cells [52]. Clustree [53] was used to display the cluster stability at different resolutions (Additional file 1: Figure S18). To visualise cell distributions, we used non-linear Uniform Manifold Approximation and Projection (UMAP) dimensionality reduction [54]. Cyclone [55] was used to estimate cell cycle stages of each cell. Pseudo-trajectory analysis was carried out with slingshot [56] using the UMAP cell projections.

We used gene regulatory network analysis independently on the two datasets—fibroblasts and iPSCs—to identify unique regulatory networks in each cell type with the pySCENIC method (v.0.10.3) using default parameters [57, 58]. The method involves three steps: (1) modules of genes that are co-expressed with transcription factors are identified from the correlation matrix using GRNboost algorithm [57]. (2) Then those modules were pruned by RcisTarget [58], a transcription factor motif enrichment analysis to identify *cis*-regulatory motifs around the putative target genes. Only genes that contain the binding motif for each respective transcription factor are retained in the module. The gene sets in these modules—known as regulons—consist of each candidate transcription factor and their target genes. (3) Lastly, the ‘activity’ of each regulon is measured in each cell using the AUCell package [58] where the area under the recovery curve (AUC) measures the activity for the regulon in that cell by calculating whether a subset of the input gene set is enriched in the expressed genes. For each regulon and cell, the AUC values were calculated using AUCell_calcAUC function, where AUC values represent the fraction of genes within the top-ranking transcription factor that were defined in each cluster. We visualised the AUCell scores generated from SCENIC with ComplexHeatmap [59].

The UMAP projected 59 cells from the fibroblast RXRB^+^ cell type in the upper right corner of the UMAP. To be sure these were not outlier cells, we tested for differentially expressed genes between the ‘central cells’ and the ‘far cells’ of this RXRB^+^ cell type but only found four genes that were differentially expressed using a Wilcox test (Additional file 1: Figure S19). SingleR [19] was used to map single cell transcriptomes against 713 reference transcriptomes.

### eQTL association analysis

To study specific regulation effects of genomic variance to gene expression, we performed statistical analysis of the association between genotypes of single nucleotide polymorphisms and single cell gene expression for 79 fibroblast cell lines and 31 iPSC cell lines generated from the same individuals. We filtered for common SNPs (minor allele frequency > 0.05) that were within ± 1 Mb of an expressed gene (detected in > 1% of the cells), resulting in 5,368,223 SNPs and 9796 genes for the fibroblasts, and 4,508,778 SNPs and 10,899 genes for the iPSCs. SNP genotypes were recoded as 0, 1 or 2 copies of the reference allele.

### Model choice

To determine the best choice of model, we tested multiple models for detecting eQTLs with the SNPs and genes located on chromosome 21. In each case, we used a linear regression to detect eQTLs but compared models with different covariates and PEER factors, and with different normalisation of the input gene expression levels [60]. In total, we tested the following linear models:

1. Gene expression calculated as the average of across cells, per cell type, per individual. Then quantile normalised and *z*-transformed without any additional covariates
2. Gene expression calculated as the average of across cells, per cell type, per individual. Then quantile normalised and *z*-transformed with one PEER factor as a covariate
3. Gene expression calculated as the average of across cells, per cell type, per individual. Then quantile normalised and *z*-transformed with ten PEER factors as covariates
4. Using all cells for a given cell type, gene expression was quantile normalised and *z*-transformed, and a random effect to account for the non-independence of the dependent variable (multiple cells from the same individual)
5. Using all cells for a given cell type with a random effect to account for the non-independence of the dependent variable (multiple cells from the same individual)

The numbers of eQTLs detected on chromosome 21 for each of these models indicated that the first three models that used the average expression per gene for each cell type per individual resulted in the identification of the largest number of eQTLs (Additional file 11: Table S10). Therefore, we further interrogated the number of PEER factors to include in the model. The final model that we selected for genome-wide eQTL detection in each cell type used the average expression level of the cells per individual for that cell type and 1 PEER factor (Additional file 1: Figure S20). PEER factors were calculated using genes that passed QC filtering (9796 genes in fibroblast cell types and 10,899 genes for iPSC cell types).

eQTL mapping was performed for each subpopulation identified by the clustering analysis. Therefore, for each subpopulation, the average expression for a given individual in that cell subpopulatipon was used to detect cis-eQTLs. *Cis*-eQTLs (SNP < 1 Mb) were detected using a linear model implemented in the MatrixEQTL R software with study-wide FDR lower than 10% using the Benjamini-Hochberg procedure [61].

### Differential expression

We used edgeR [62, 63] to identify differentially expressed genes between each cell type compared with the other cell types combined (i.e. each fibroblast type compared to the other five fibroblast types and each iPSC type compared to the other three iPSC types). Differentially expressed genes were detected using the gene-wise negative binomial generalised model with a quasi-likelihood test. Detection rate and pool batches were included as covariates following the recommendations of Soneson and Robinson [31]. Heatmaps and upset plots were generated using ComplexHeatmap [59] in R. Heatmaps were created with scaled, normalised data.

### Independent eQTL analysis

Given an eGene that was significant in a pair of cell types (*a* and *b*), the top eSNPs from each cell type (*S*_*a*_ and *S*_*b*_) were tested for independence with relation to eGene expression. Accordingly, the top eSNP (*S*_*b*_) in cell type *b* was regressed from the linear model for the association of the top eSNP, *S*_*a*_, for cell type *a* with gene expression of the eGene (*G*_*a*_) in cell type *a*.

$$ {G}_a\sim {\beta}_0+{\beta}_1{S}_a+{\beta}_2{S}_b+{PEER}_1+\varepsilon $$

eSNPs were deemed independent if the association between *S*_*a*_ and *G*_*a*_ was significant following regression of *S*_*b*_ in the linear model. This model works well for these data since all the scRNA-seq data for fibroblast and iPSC cells was generated, processed and underwent quality control assessment together.

### Interaction eQTL analysis

Given an eGene that was significant in at least two cell types, the eSNP with the largest difference between their beta allelic effects between any two clusters was used to test for cell type interaction. Two models were fit for gene expression *G*, with SNP *S* and cell type *C*. The first model (1) was a normal linear model and the second model (2) included an interaction term. An interaction was considered significant if an *anova* comparing the two models was significant.

1. *G*~*β*_0_ + *β*_1_*S* + *β*_2_*C* + *PEER*_*1*_ + β
2. *G*~*β*_0_ + *β*_1_*S* + *β*_2_*C* + *β*_3_*SC* + *PEER*_*1*_ + β

### eGene correlation

The expression of eGenes that were unique to a given cell type were correlated with their expression in the other cell types using a *Pearson* correlation test.

### Test statistic correlation

The significant eQTL test statistics from each cell subtype was compared with test statistic for the same eGene-eSNP pair in all other cell types to test whether one contributing factor for the highly cell type-specific eQTL detection could be due to lack of power.

### eGene enrichment

eGenes from a specific cell type were tested for enrichment in the other cell types. eGenes were ranked based on the lowest *p* value for each eGene. An expected distribution of mean rank scores was generated from 10,000 permutations of randomly selected genes (selecting the same number of genes as eGenes). The mean rank of the eGenes in the testing cell types was then tested for significance with a *t* test.

### GTEx comparison

Gene Tissue Expression (GTEx) [22] database version seven results were downloaded on 6 July 2019. The cultured fibroblast cell eQTL were compared with the fibroblast cell type eQTL results to identify common and unique results.

### iPSC reprogramming comparison

Genes differentially expressed during iPSC reprogramming from fibroblasts were obtained from Liu et al. [32]. Gene IDs were used to identify reprogramming genes that were also eQTLs. Differentially methylated regions (DMRs) between fibroblasts and iPSCs were obtained from Lister et al. [33] and were checked for overlapping eSNPs. DMRs between iPSCs and ESCs were obtained from Polo et al. [34] and were checked for eSNPs within 100 bp of the differentially methylated base pair.

### Figure preparation and creation

Plots panels were prepared in R with ggplot [64], Gviz [65], ComplexHeatmap [59] and LDlinkR [66]. They were arranged and edited with BioRender.com.

## Supplementary Information

**Additional file 1: Figure S1-S20**.

**Additional file 2: Table S1**. Top 50 transcription factors regulating fibroblast cell types.

**Additional file 3: Table S2**. Top 50 transcription factors regulating iPSC cell types.

**Additional file 4: Table S3**. Differentially expressed genes in specific fibroblast types.

**Additional file 5: Table S4**. Differentially expressed genes in specific iPSC types.

**Additional file 6: Table S5**. Significant fibroblast type eQTLs.

**Additional file 7: Table S6**. Significant iPSC type eQTLs.

**Additional file 8: Table S7**. Transcription factor binding motifs that are interrupted by eQTL SNPs.

**Additional file 9: Table S8**. eGenes with independent top eQTLs in different cell types.

**Additional file 10: Table S9**. eQTLs that demonstrate SNP-cell type interactions.

**Additional file 11: Table S10**. eQTL models tested.

**Additional file 12:.** Review history.

## Data Availability

The scRNA-seq data for all 79 fibroblast cell lines and 31 iPSC cell lines are available from ArrayExpress (Accession Number: E-MTAB-10060) [67].
