# Supplementary material for: Single cell eQTL analysis identifies cell type-specific genetic control of gene expression in fibroblasts and reprogrammed induced pluripotent stem cells
Source: Genome Biol. 2021 Mar 5;22:76. doi: 10.1186/s13059-021-02293-3 (PMC7934233; doi:10.1186/s13059-021-02293-3)
Supplement: Supplementary file 1 — Additional file 1: Figure S1-S20. [file 13059_2021_2293_MOESM1_ESM.docx]

**Single cell eQTL analysis identifies cell-type specific genetic control of gene expression in fibroblasts and reprogrammed induced pluripotent stem cells**

Drew Neavin^1*^, Quan Nguyen^2*^, Maciej S. Daniszewski^3,4,5^, YongKiat WeeDaniel Wee^1^, Helena H. Liang^3,4^, Han Sheng Chiu^2^, Anne Senabouth^1^, Samuel W Lukowski^2^, Duncan E. Crombie^3,4^, Grace E. Lidgerwood^3,4,5^, Damián Hernández^3,4,5^, James C. Vickers^6^, Anthony L. Cook^6^, Nathan J. Palpant^2,#^, Alice Pébay^3,4,5,#^, Alex W. Hewitt^3,4,7,#^ , Joseph E. Powell^1,8,9,#^

**SUPPLEMENTARY FIGURES**

**
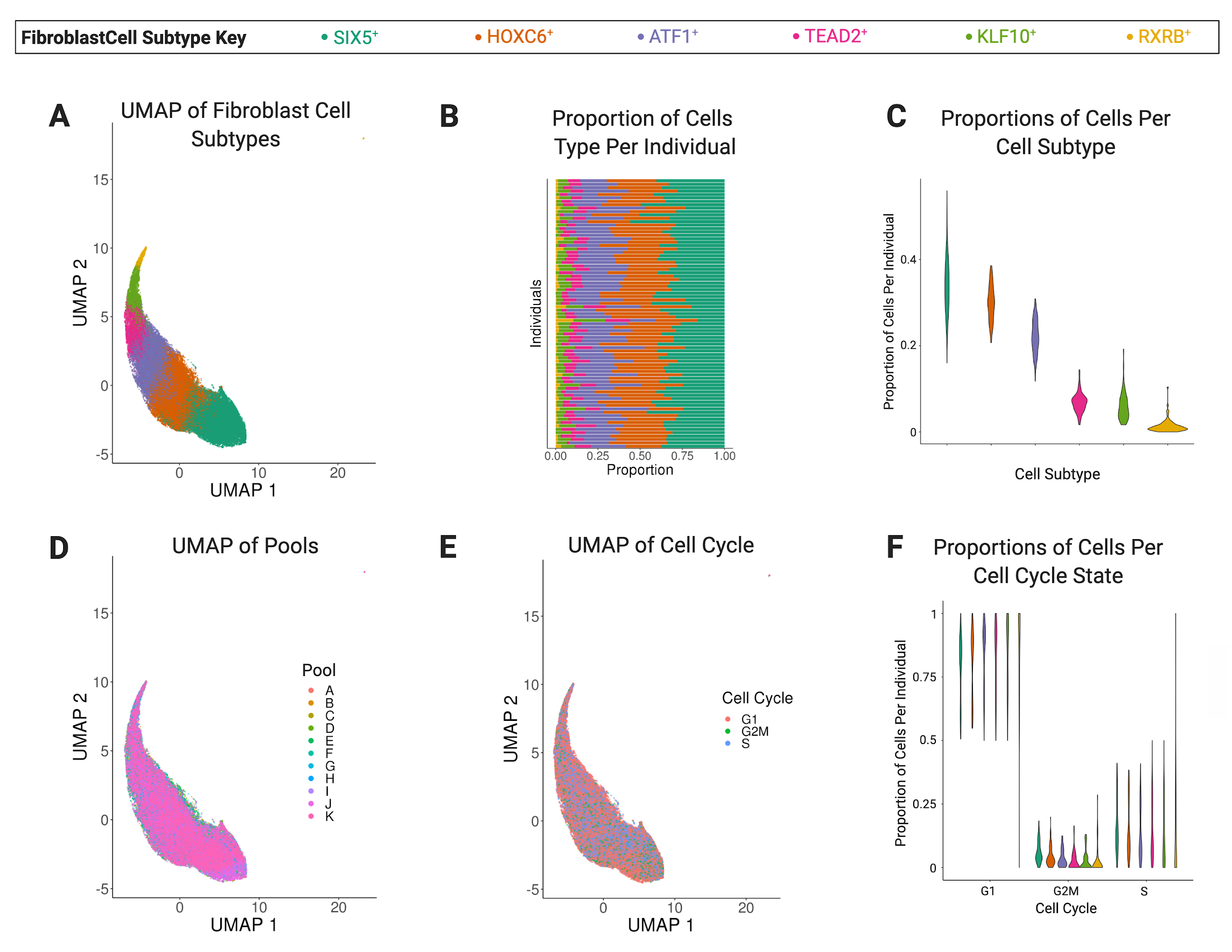
 Figure S1: Quality Control Metrics of Fibroblast Subtypes. A)** Six subtypes of fibroblasts were identified. **B-C)** The proportions of each fibroblast subtype were consistent across individuals. **D)** The 10x capture pools were evenly distributed across the six fibroblast subtypes. **E-F)** Cell cycle states were also evenly distributed across fibroblast subtypes.


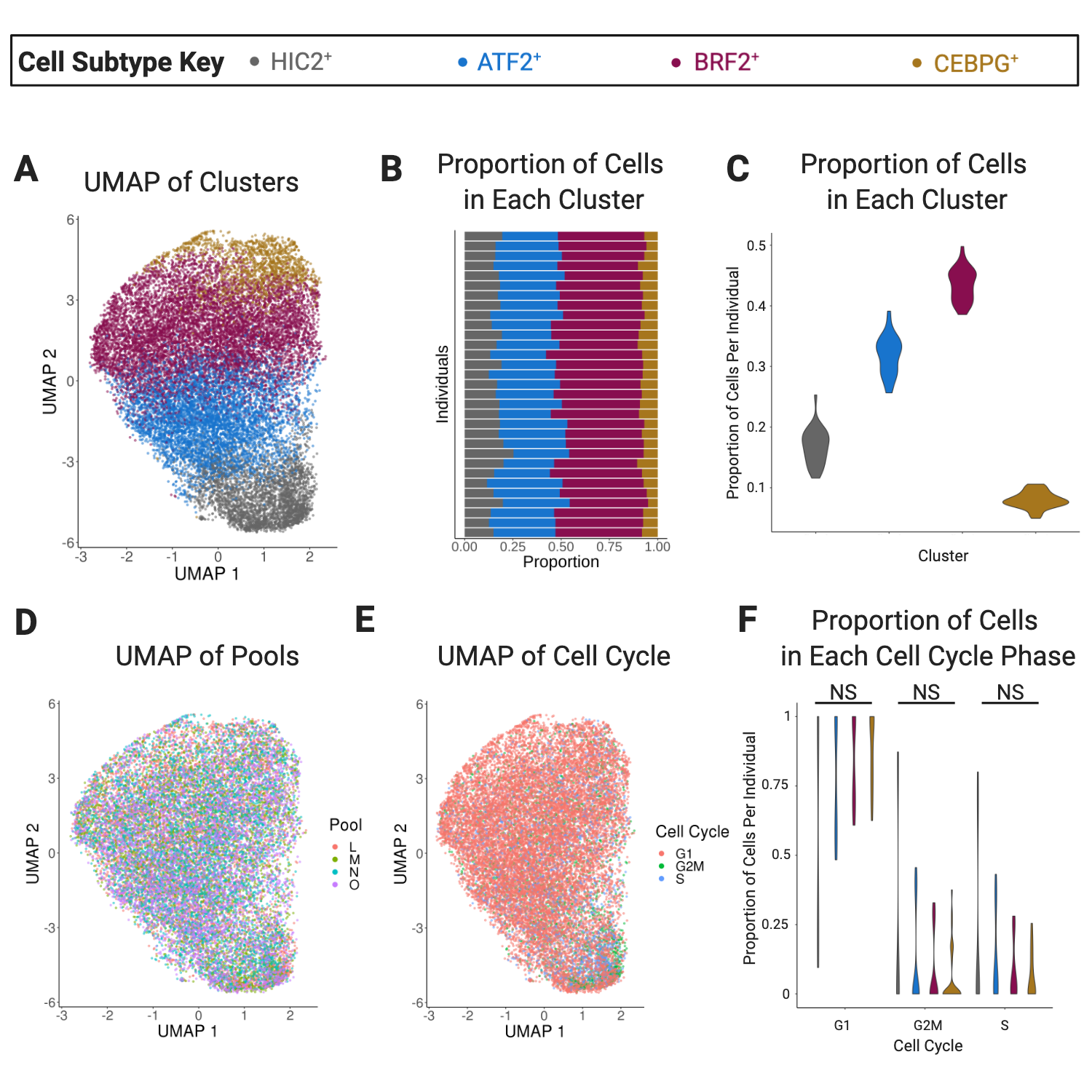


**Figure S2: Quality Control Metrics of Fibroblast Subtypes. A)** Four subtypes of induced pluripotent stem cells (iPSCs) were identified. **B-C)** The proportions of each iPSC subtype were consistent across individuals. **D)** The 10x capture pools were evenly distributed across the six iPSC subtypes. **E-F)** Cell cycle states were also evenly distributed across iPSC subtypes which was tested with a Kruskal-Wallis test.

**
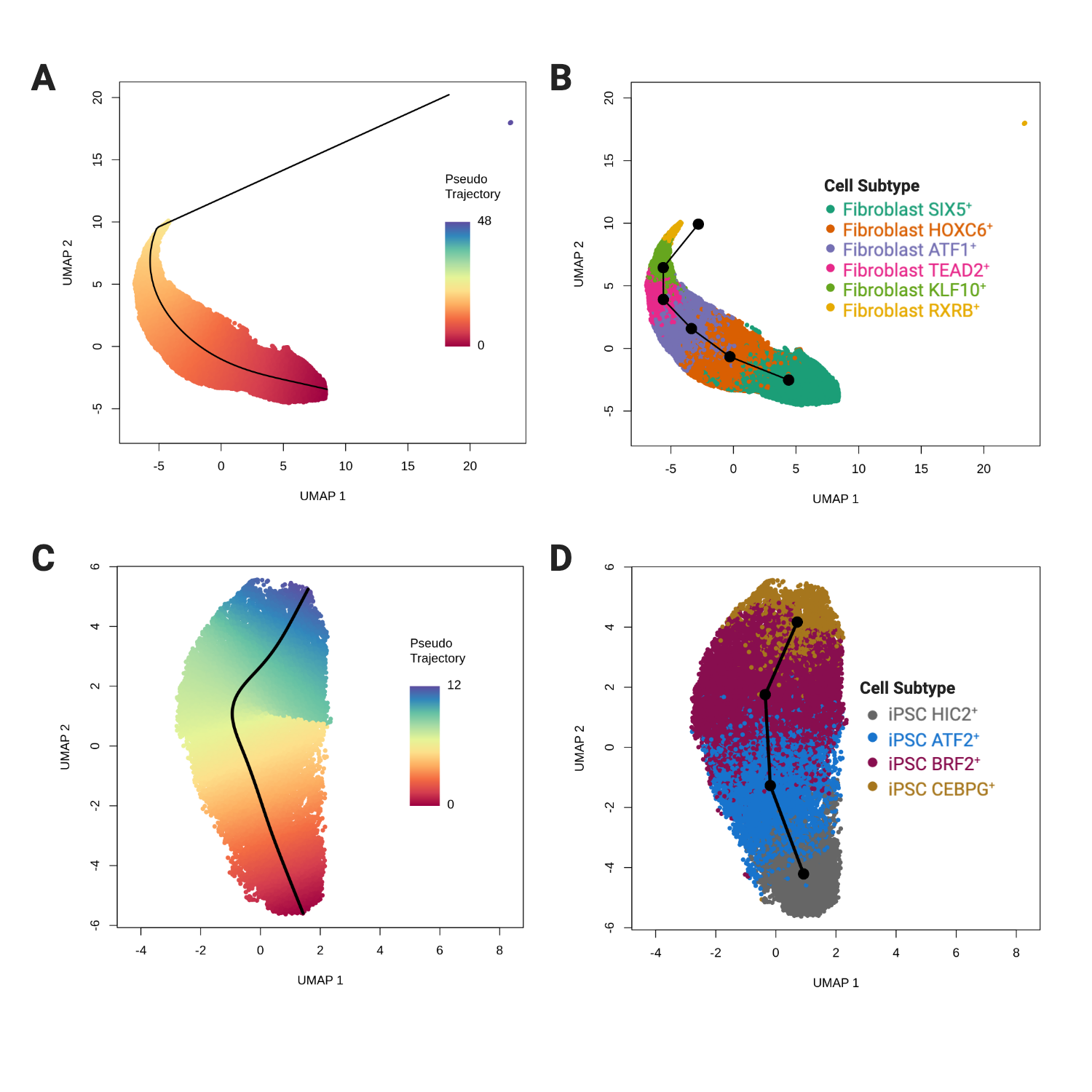
**

**Figure S3: Fibroblast and iPSC Pseudotrajectories. A)** Pseudotrajectory across fibroblast single cells colored by the slingshot pseudotrajectory values B) Fibroblast subtype pseudotrajectory colored by the fibroblast subtype. C) Pseudotrajectory across iPSC single cells colored by slingshot pseudotrajectory values. D) iPSC subtype pseudotrajectory colored by the iPSC subtypes. iPSC: induced pluripotent stem cell.


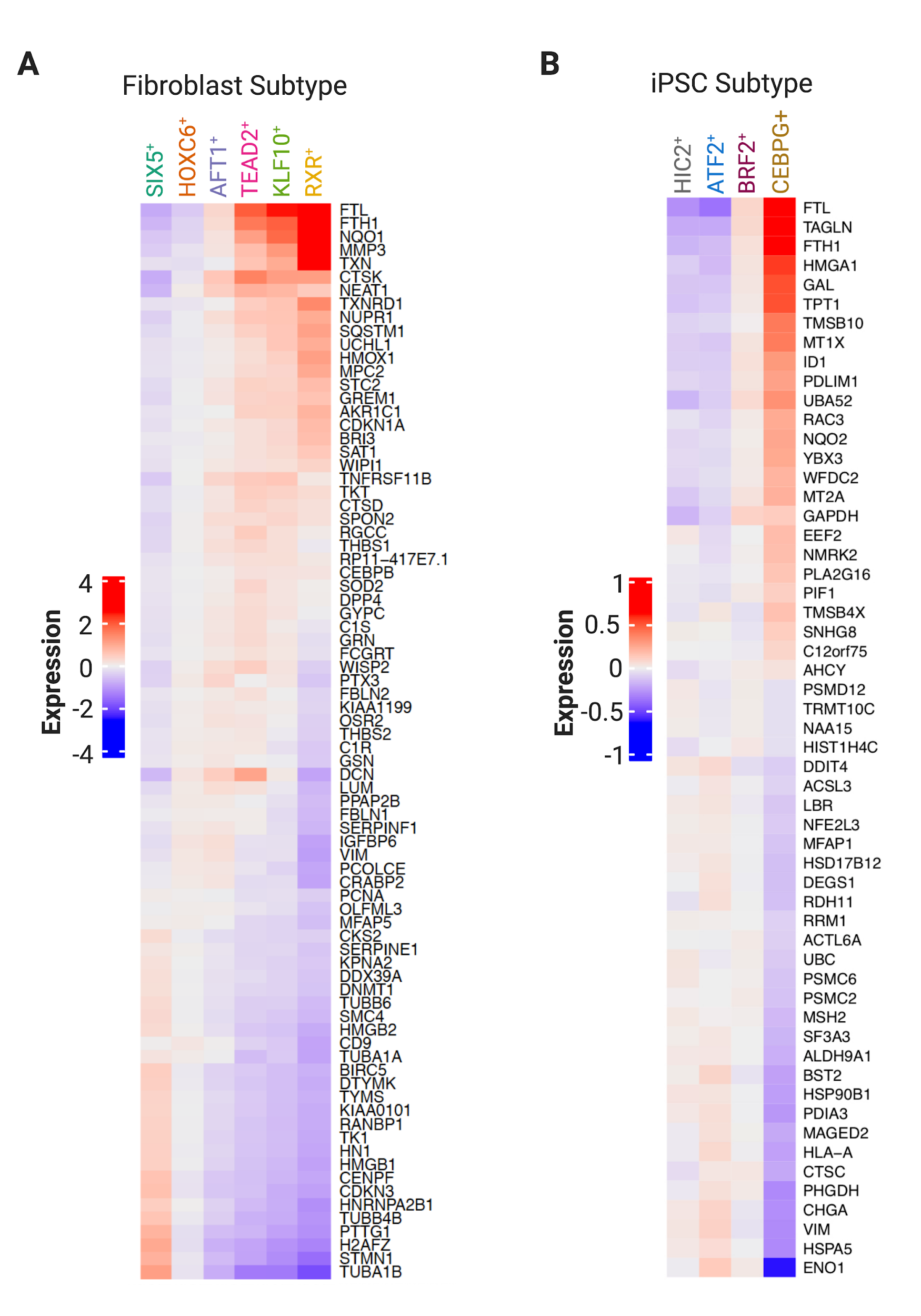


**Figure S4: Top 20 differentially expressed genes across fibroblast and iPSC subtypes. A)** Expression of top 20 differentially expressed genes across the six fibroblast subtypes. **B)** Expression of top 20 differentially expressed genes across the four iPSC subtypes. iPSC: induced pluripotent stem cell.

**
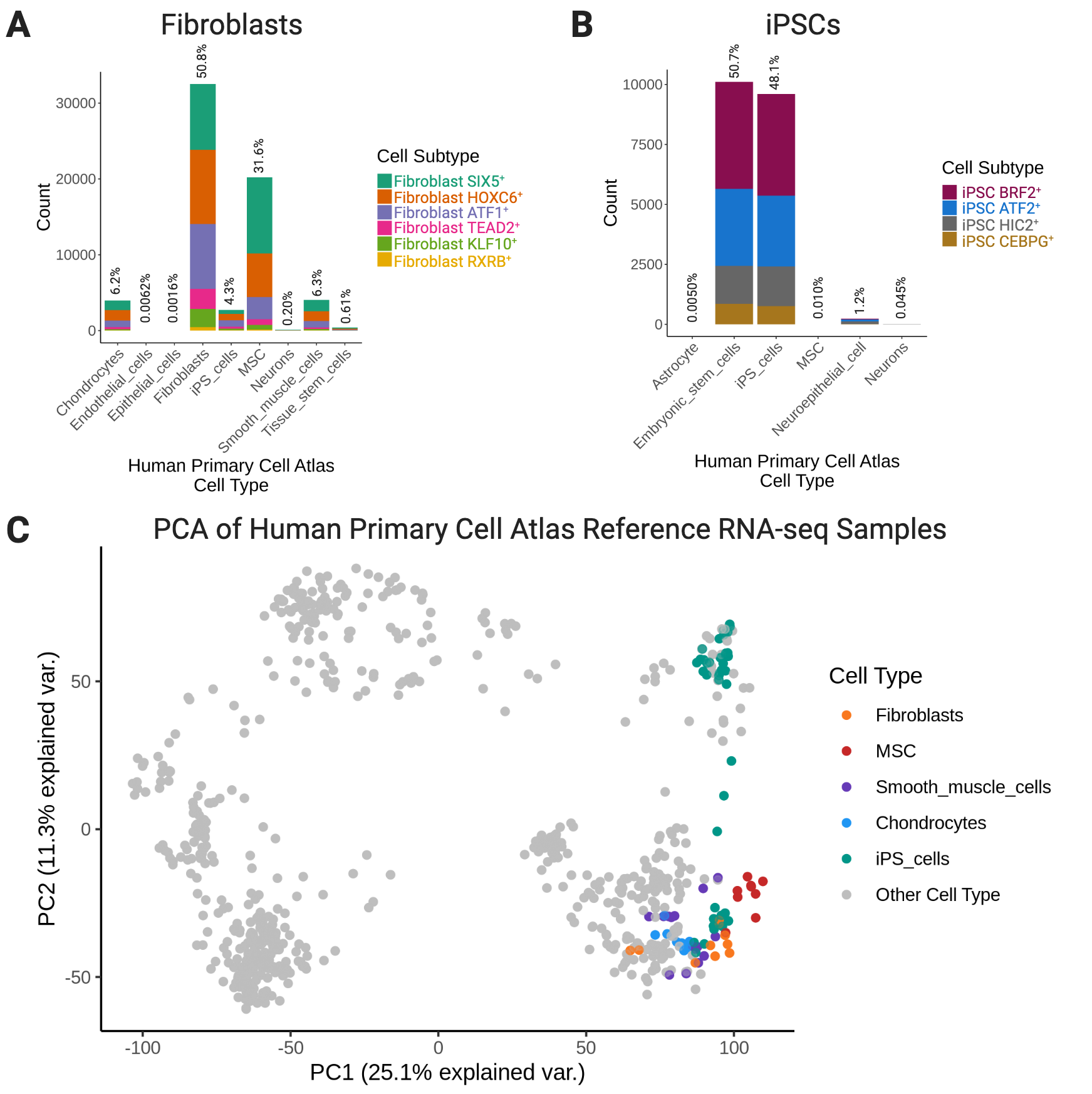
Figure S5: Map scRNA-seq Transcriptomes to Reference Datasets. A)** The transcriptional profiles of fibroblast cells were mapped to 713 reference transcriptomes available from the human primary cell atlas. The majority of these cells were mapped to the Fibroblast reference dataset. **B**) iPSC scRNA-seq transcriptional profiles were mapped to 713 reference transcriptomes from the human primary cell atlas. The vast majority of iPSC single cell transcriptional profiles were mapped to stem cells (embryonic or induced pluripotent stem cells). **C**) The reference cell types that >4% of fibroblast cells were assigned to demonstrate similar transcriptional profiles - visualized by the top 2 principal components which cumulatively account for 36.4% of the variance. iPSC: induced pluripotent stem cell; MSC: mesenchymal stem cell.


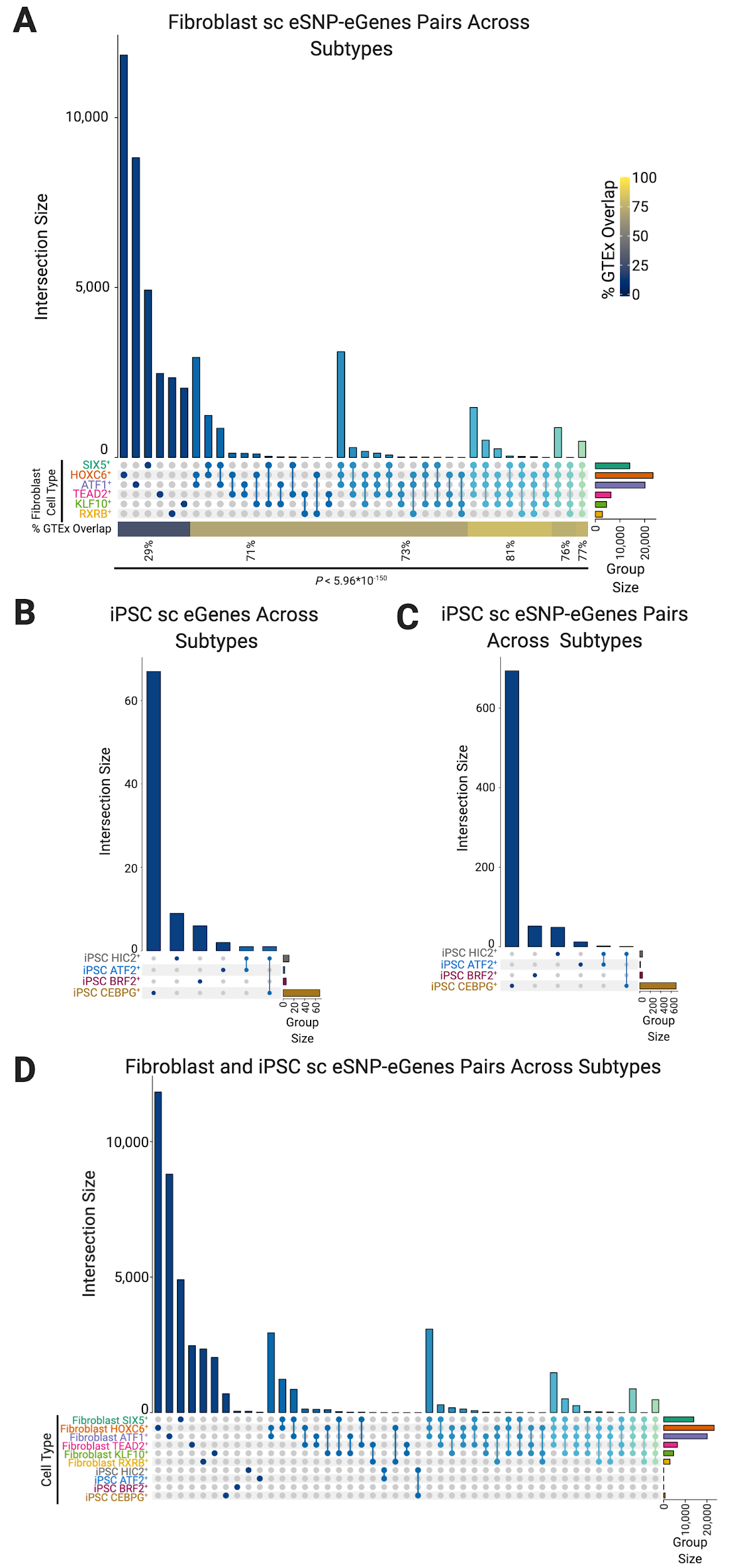


**Figure S6: Overlap of sc eQTLs in Fibroblasts and iPSCs.** **A)** The majority of eSNP-eGene pairs in fibroblast subpopulations are unique to a given subpopulation. Further, the percent of those sc eSNP-eGene pairs that were detected in GTEx transformed fibroblasts increased with increasing numbers of fibroblast subtypes that they were detected in (P = 1E-108, Cochran-Armitage Test). **B)** The majority of iPSC eGenes were unique to an iPSC subtype and none were significant in more than two subtypes. **C)** In addition, the majority of iPSC eSNP-eGene pairs were also unique to a single iPSC subtype. **D)** No eSNP-eGene pair was significant in both fibroblast and iPSC subtypes. **E)** However, some eGenes were significant in both fibroblast and iPSC subtypes. eSNP: eQTL SNP; eGene: eQTL gene; eQTL: expression quantitative trait locus; iPSC: induced pluripotent stem cell.

**
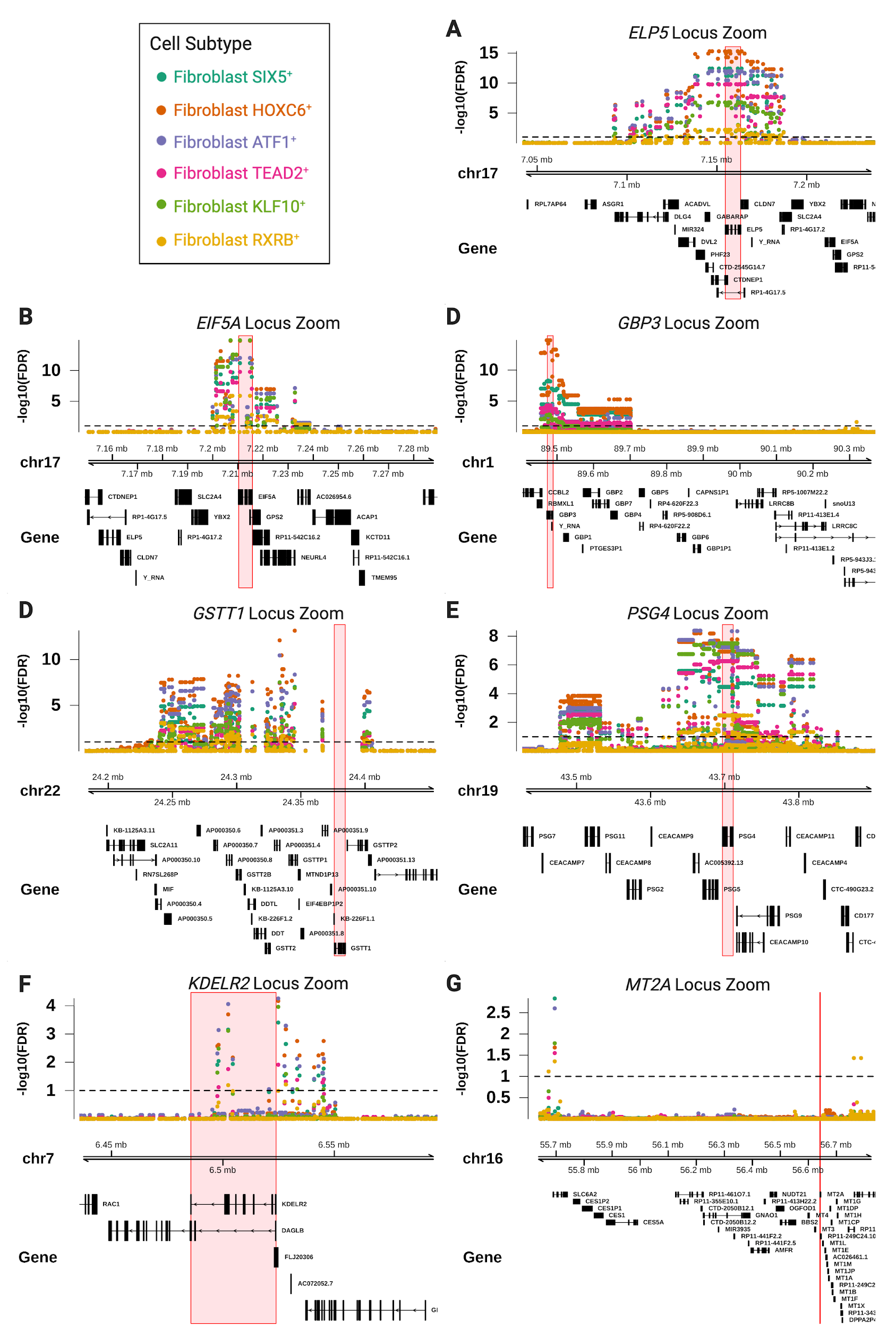
**

**Figure S7: eGenes that were significant in all six fibroblast subtypes.** Eight eGenes were significant in all six fibroblast subtypes. Those included *ELP5* (**A**), *EIF5A* (**B**), *GBP3* (**C**), *GSTT1* (**D**), *PRKCDBP* (**E**), *PSG4* (**F**), *KDELR2* (**G**) and *MTA2* (**H**).
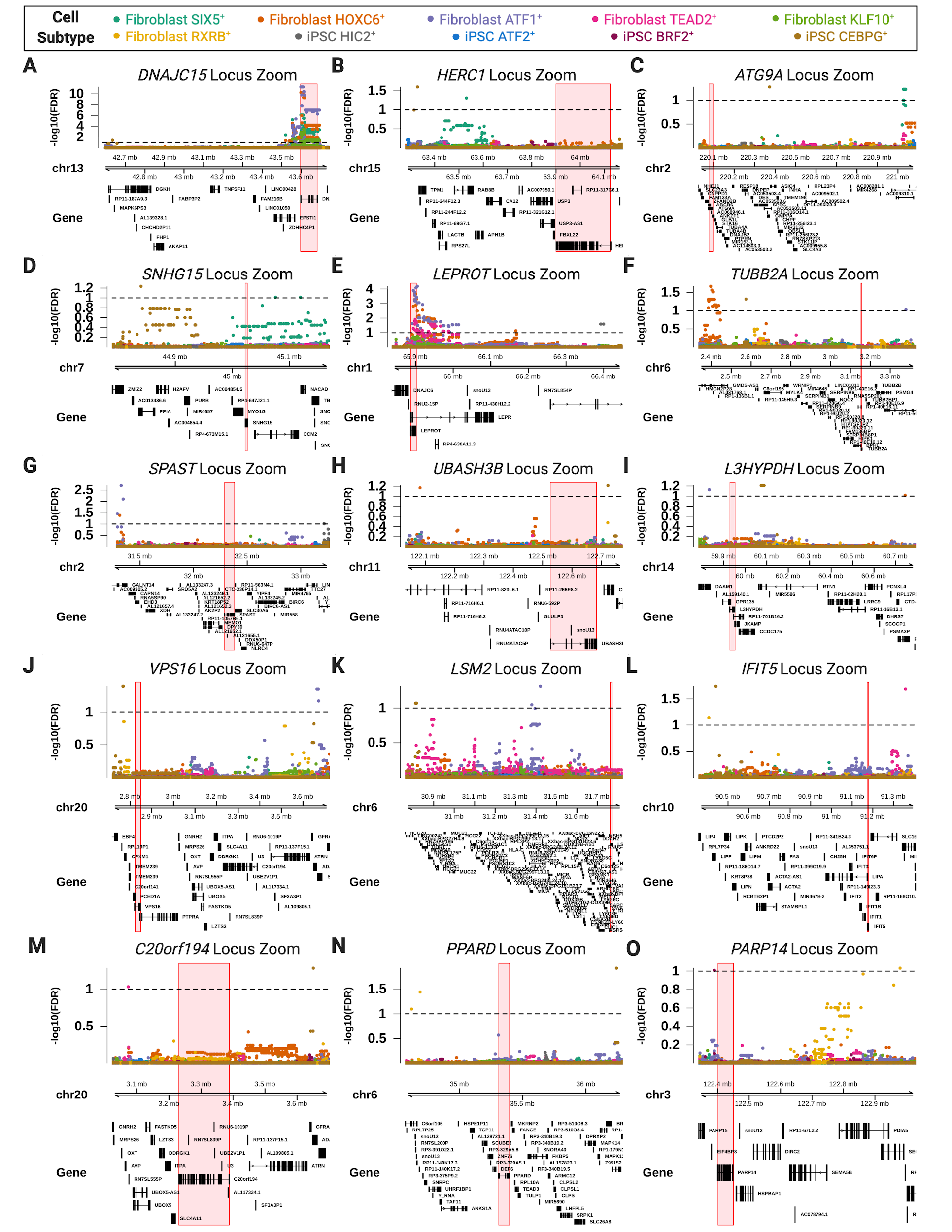


**Figure S8: eGenes that were Significant in both iPSC and Fibroblast Subtypes.** Eleven eGenes were significant in at least one iPSC and one fibroblast subtype. Those included *PRKD3* (**A**), *ATG9A* (**B**), *MIOS* (**C**), *GEMIN5* (**D**), *IFIT5* (**E**), *TCP11L1* (**F**), *EXT1* (**G**), *ARSK* (**H**), *CRTC1* (**I**), *PPARD* (**J**) and *SPAST* (**K**). In all cases, the eSNPs for the fibroblast and iPSC subtypes were either on opposite sides of the gene or separated by at least 400,000 base pairs. iPSC: induced pluripotent stem cell.


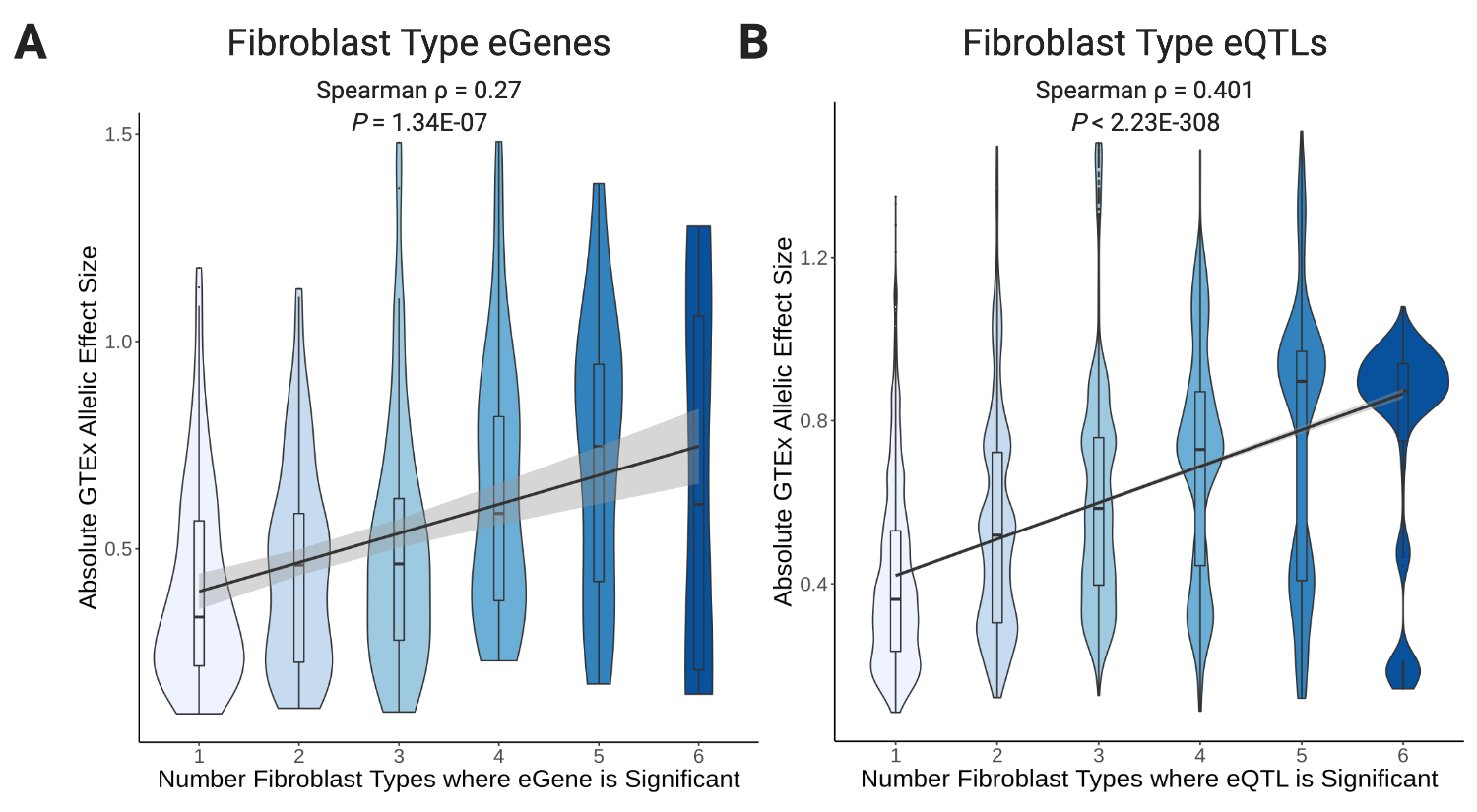


**Figure S9: Absolute GTEx Cultured Fibroblast Allelic Effect Sizes by Number of Significant Fibroblast Types.** **A)** The absolute allelic effect size from GTEx cultured fibroblasts is significantly correlated with the number of fibroblast types where that eGene was significant. The absolute allelic effect size in GTEx cultured fibroblasts was smallest for those eGenes that were significant in just one fibroblast type. **B)** The absolute allelic effect size from GTEx cultured fibroblasts is significantly correlated with the number of fibroblast types where that eQTL was significant. The absolute allelic effect size in GTEx cultured fibroblasts was smallest for those eQTLs that were significant in just one fibroblast type.

**
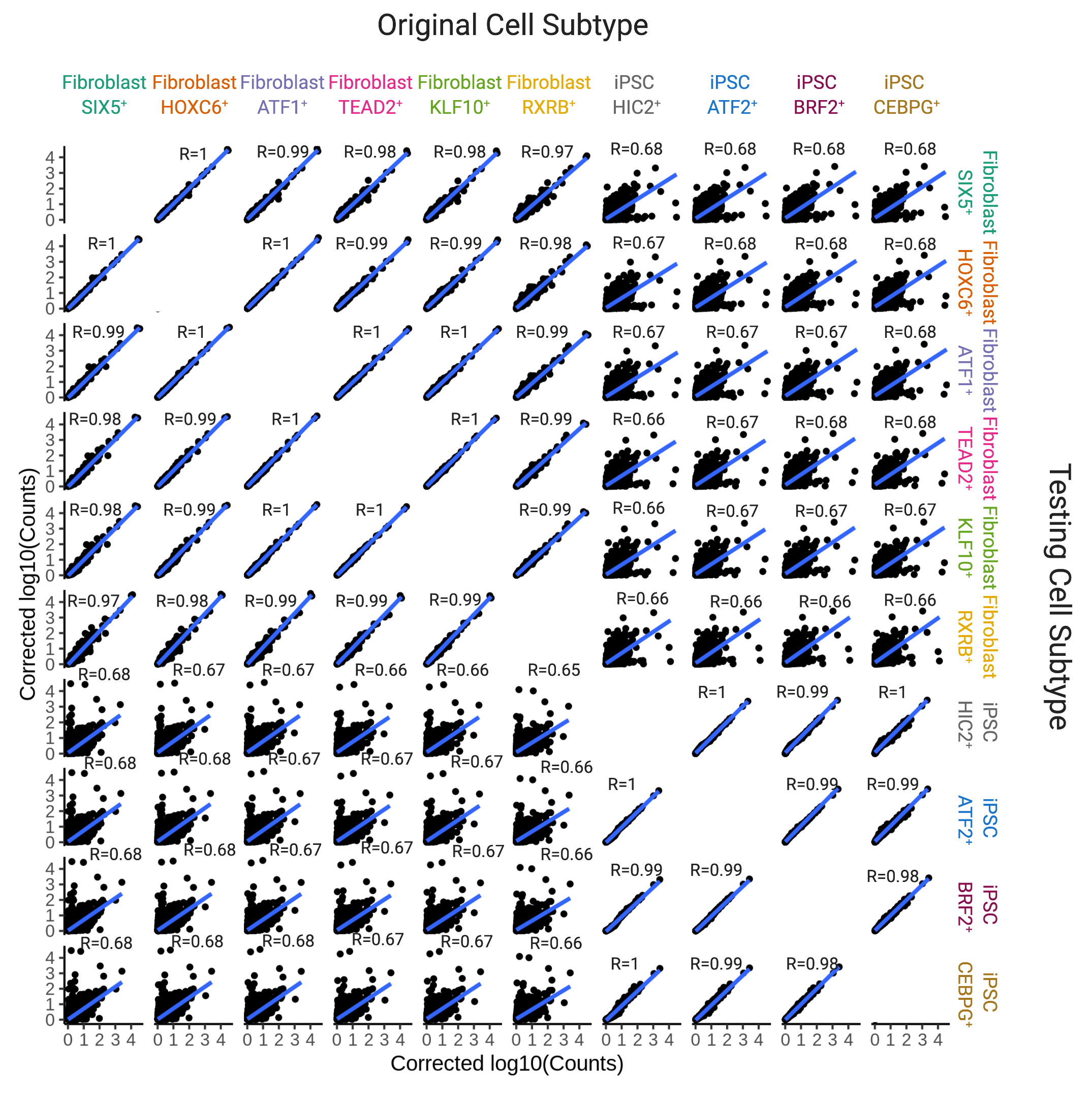
Figure S10: Correlation of eGene Expression.** eGene expression from the one cell subtype (“Original Cell Subtype”) were correlated for expression in the other cell subtypes (“Testing Cell Subtype”). Pearson correlation was used to test the linear relationship of the “Original Cell Subtype” eGene expression with expression in the “Testing Cell Subtype”. eGene: eQTL gene; iPSC: induced pluripotent stem cell.


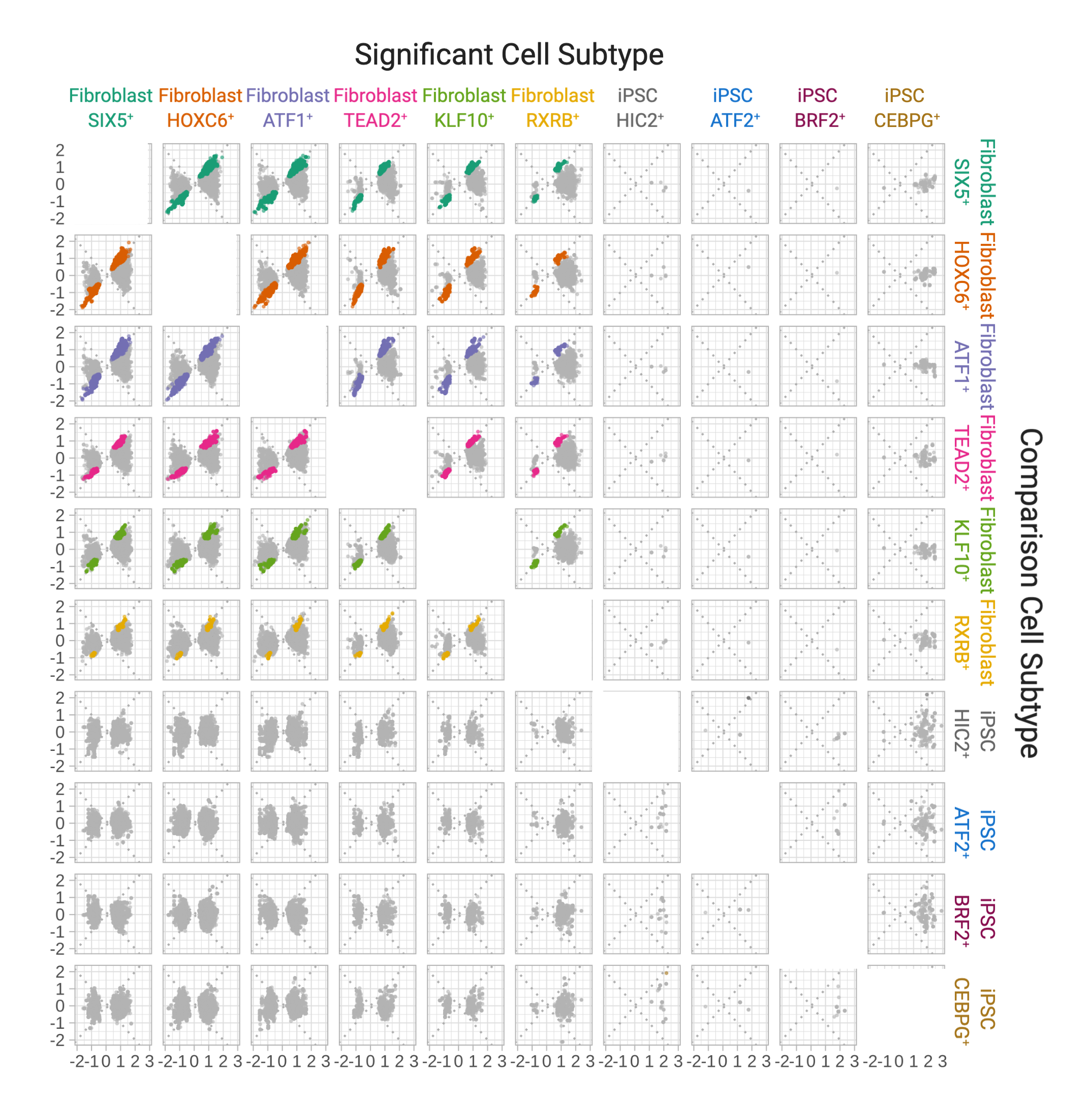


**Figure S11: Correlation of eQTL Test Statistics Across Cell Types.** The significant eQTLs from each cell type were compared to the test statistic in the other cell types and in most cases, those that were well correlated were already significant in the other cell type (colored points) but those that did not demonstrate strong correlation between the test statistics in the two different cell types were not significant (grey points). eQTL: expression quantitative trait locus; iPSC: induced pluripotent stem cell.


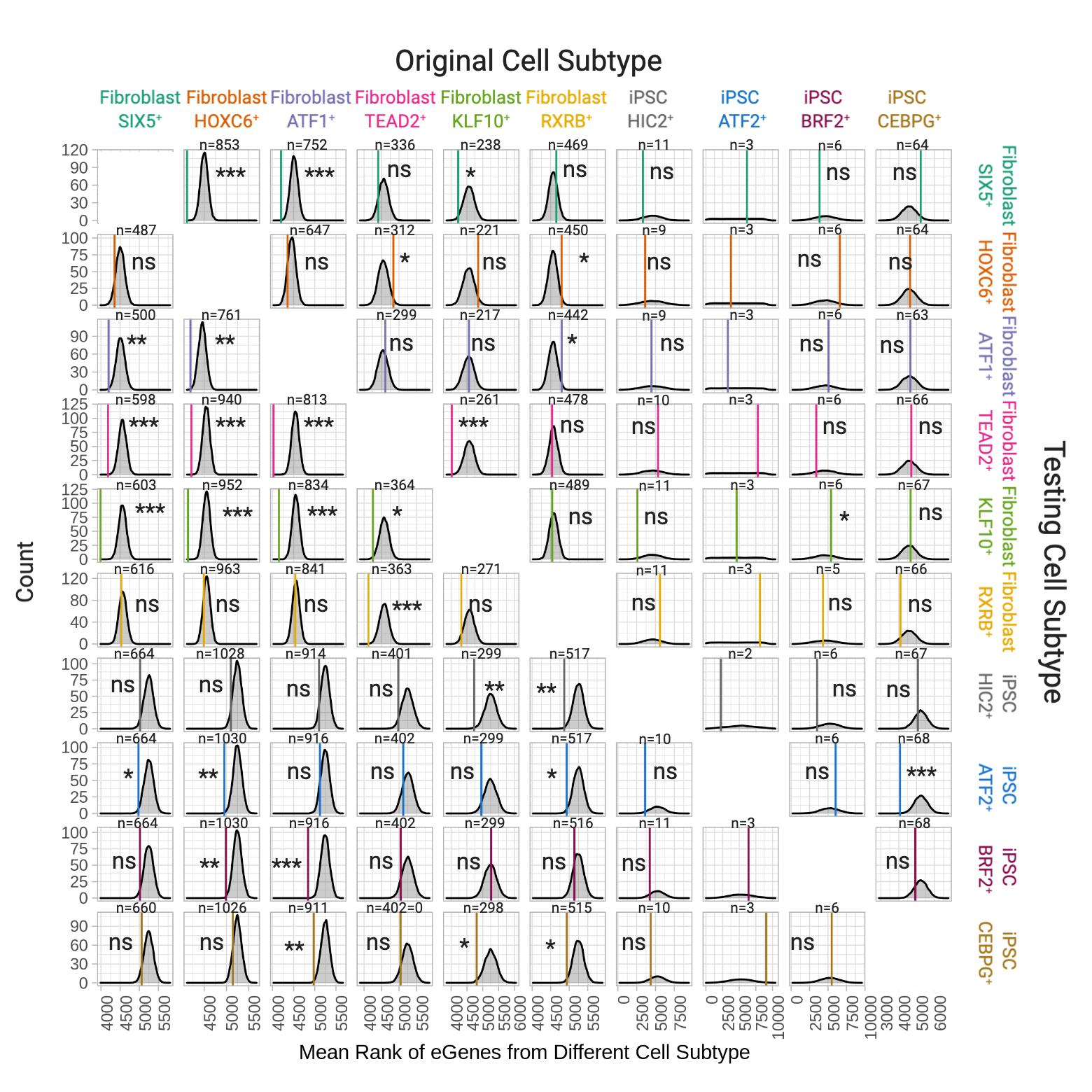


**Figure S12: Enrichment of Unique Significant eGenes in other Subtypes.**

Significant eGenes from each cell subtype were tested for enrichment in other cell subtypes. The grey densities represented 10,000 permutations of mean rank of randomly-selected eGenes, the colored line is the mean rank of the eGenes from the original cell subtype in the testing cell subtype. The iPSC FTL^lo^/BST2^hi^ cell subtype had too few unique eGenes (1) to test for enrichment in other cell subtypes. A student’s t-test was used to test if the mean rank of the eGenes was significantly different from the genes randomly selected from the testing cell subtype eGene ranked list. **P* < 0.05; ***P* < 0.01; ****P* < 0.001; ns=non-significant. eGene: eQTL gene; iPSC: induced pluripotent stem cell.


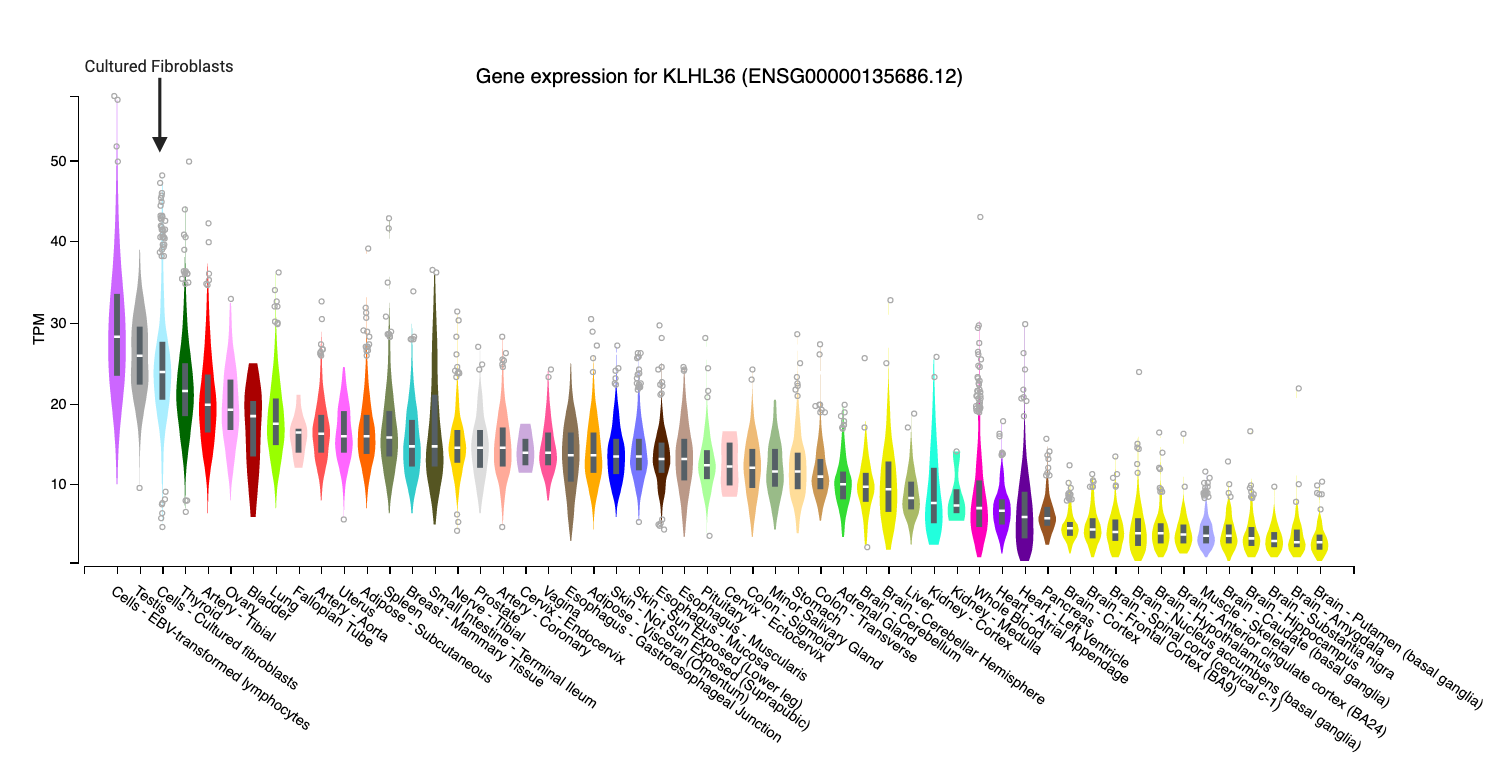


**Figure S13: *KLHL36* Gene Expression in GTEx.** KLHL36 is highly expressed in cultured fibroblasts compared to most other tissue types included in the GTEx database. GTEx: Genotype tissue expression.


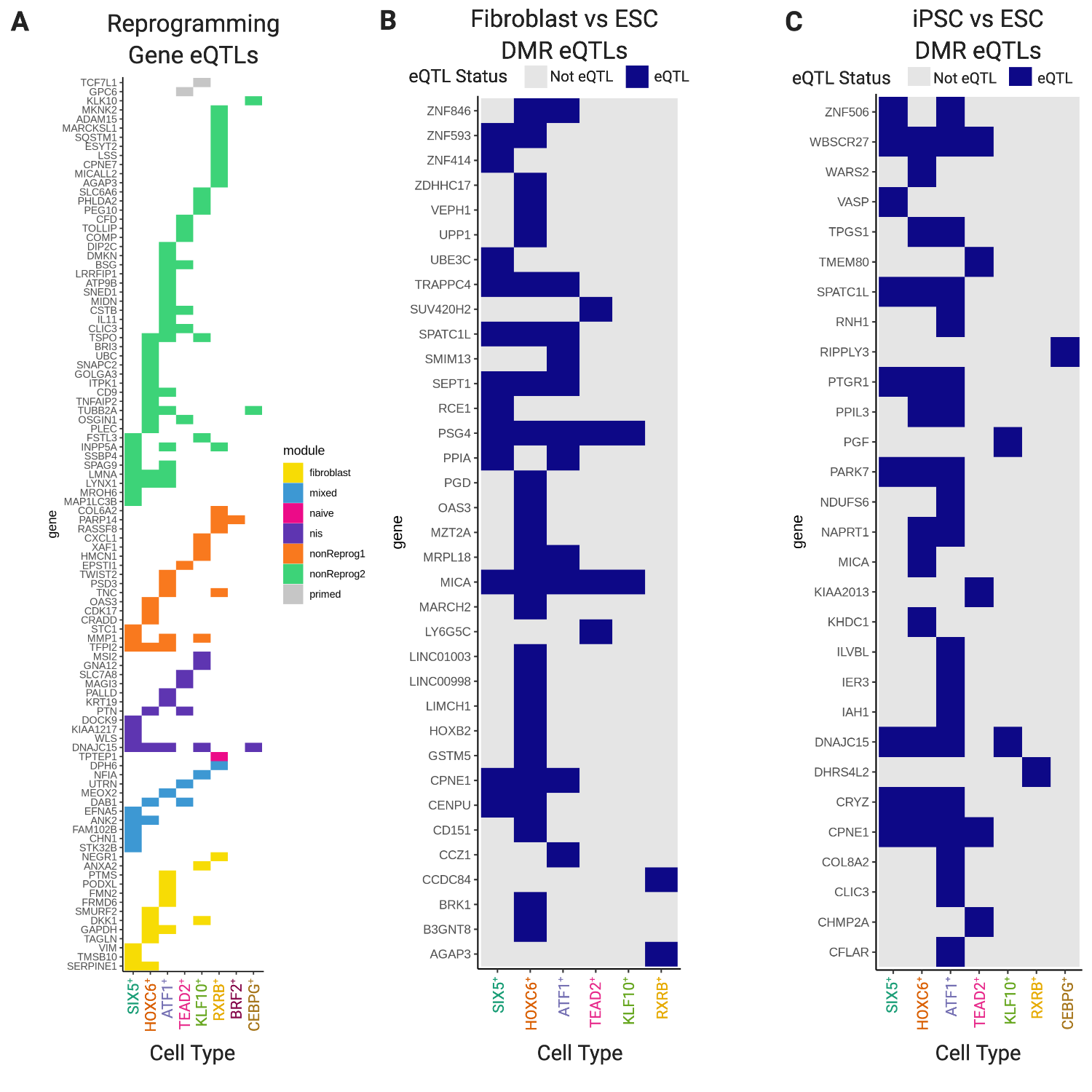


**Figure S14: eQTLs in genes that characterize iPSC reprogramming. A)** The genes that characterize cell types during reprogramming have eQTLs - the majority of which are cell type specific (76.5%). **B)** Genomic regions differentially methylated between fibroblasts and iPSCs - indicating important regions for reprogramming - contain many cell type specific eQTLs (68.8%). **C)** The majority of eQTLs in regions differentially methylated between iPSCs and ESCs - regions that may indicate incomplete reprogramming - are cell type specific (62.1%). DMR: differentially methylated region; eQTL: expression quantitative trait locus; ESC: embryonic stem cell.


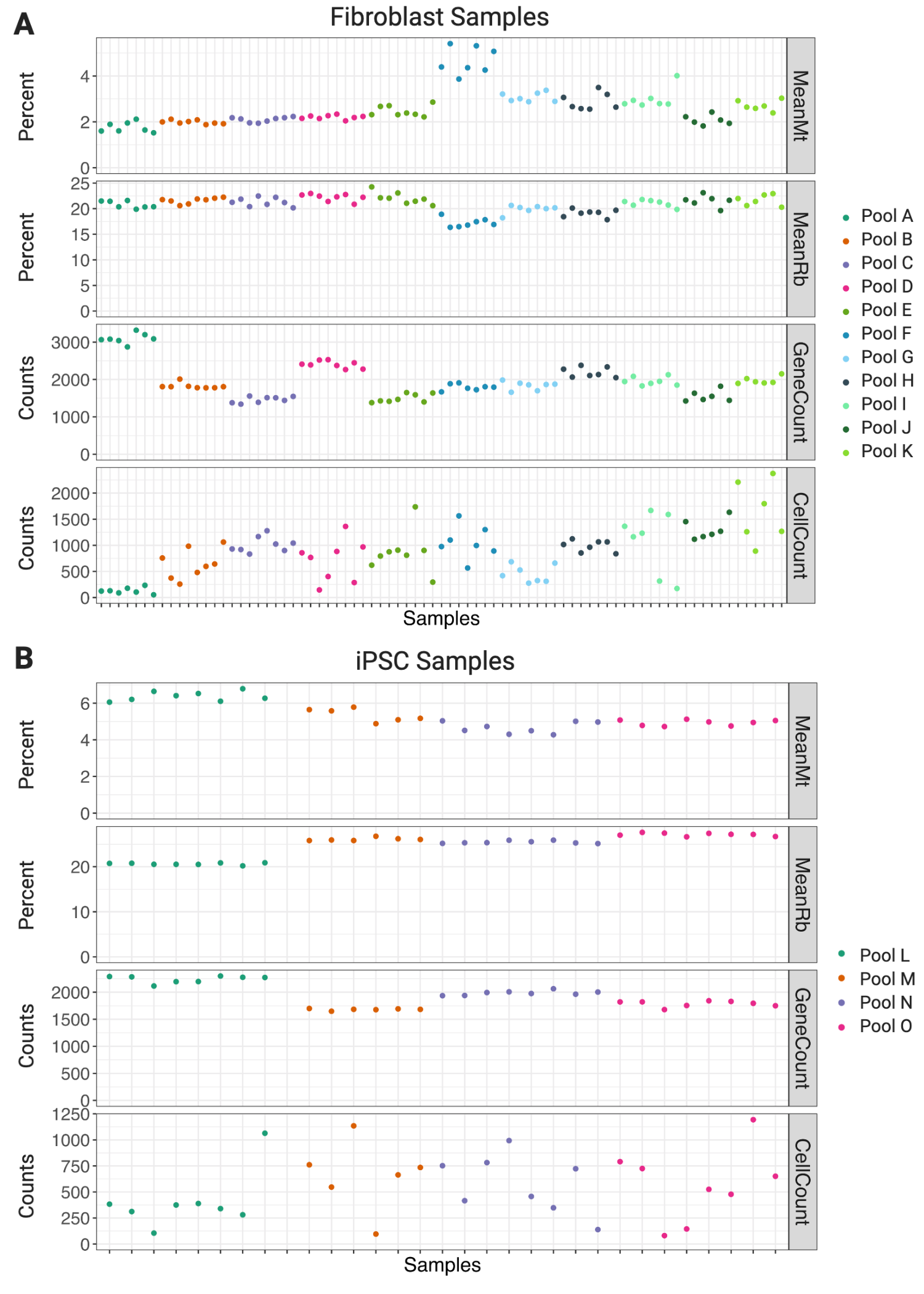


**Figure S15: Average Cell Quality Measures by Sample.** Mean percent of unique reads that map to mitochondrial genes (MeanMt), mean percent of unique reads that map to ribosomal genes (MeanRb), mean number of genes detected per cell and mean number of cells per sample before quality control processing in fibroblast (**A**) and iPSC (**B**) samples. iPSC: induced pluripotent stem cell


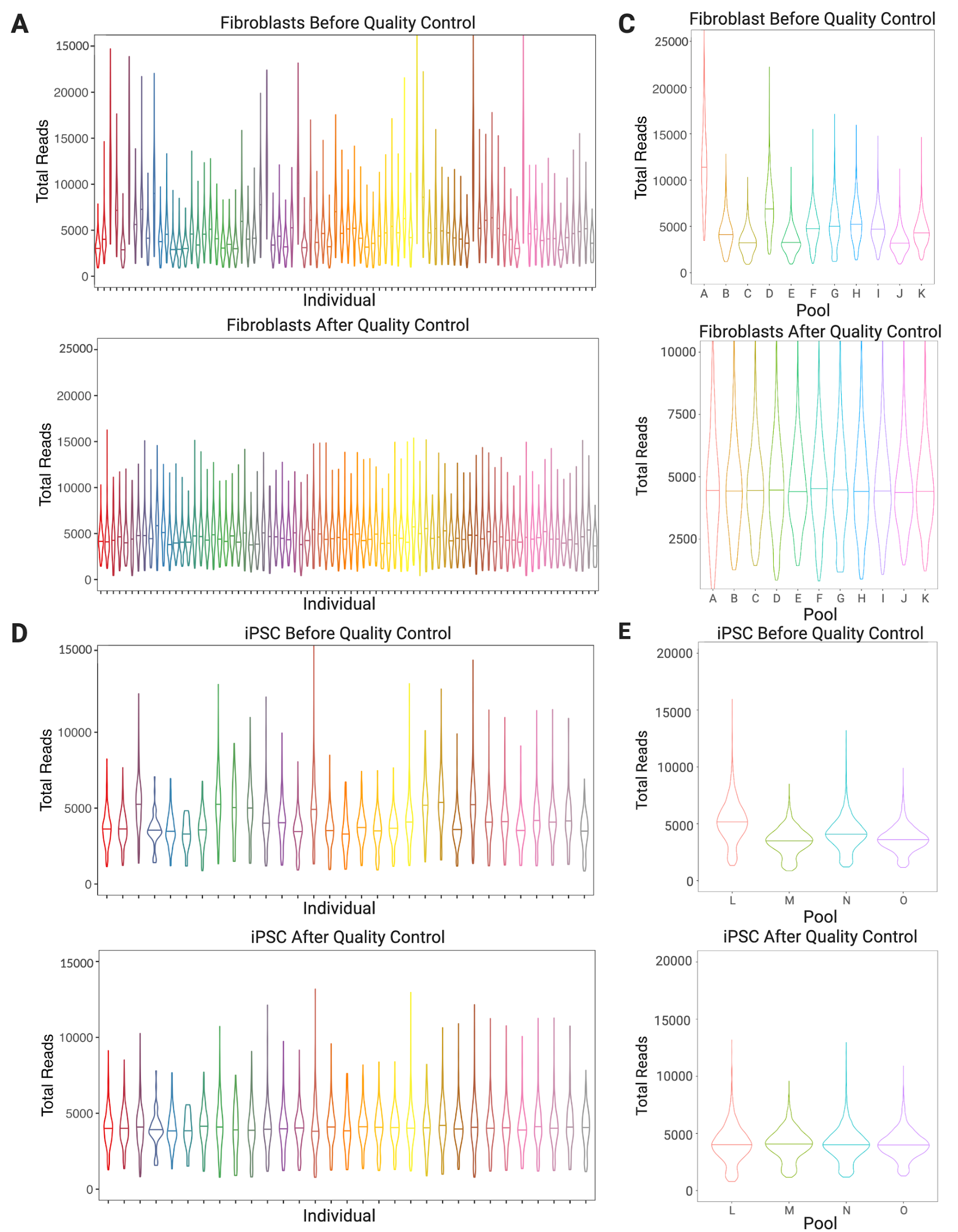


**Figure S16: Total Reads per Cell Before and After Quality Control Filtering.** Quality control filtering resulted in consistent distributions of the total number of reads per cell across individuals in fibroblasts (**A**) and iPSC (**B**) as well as across pools in fibroblasts (**C**) and iPSC (**D**). iPSC: induced pluripotent stem cell


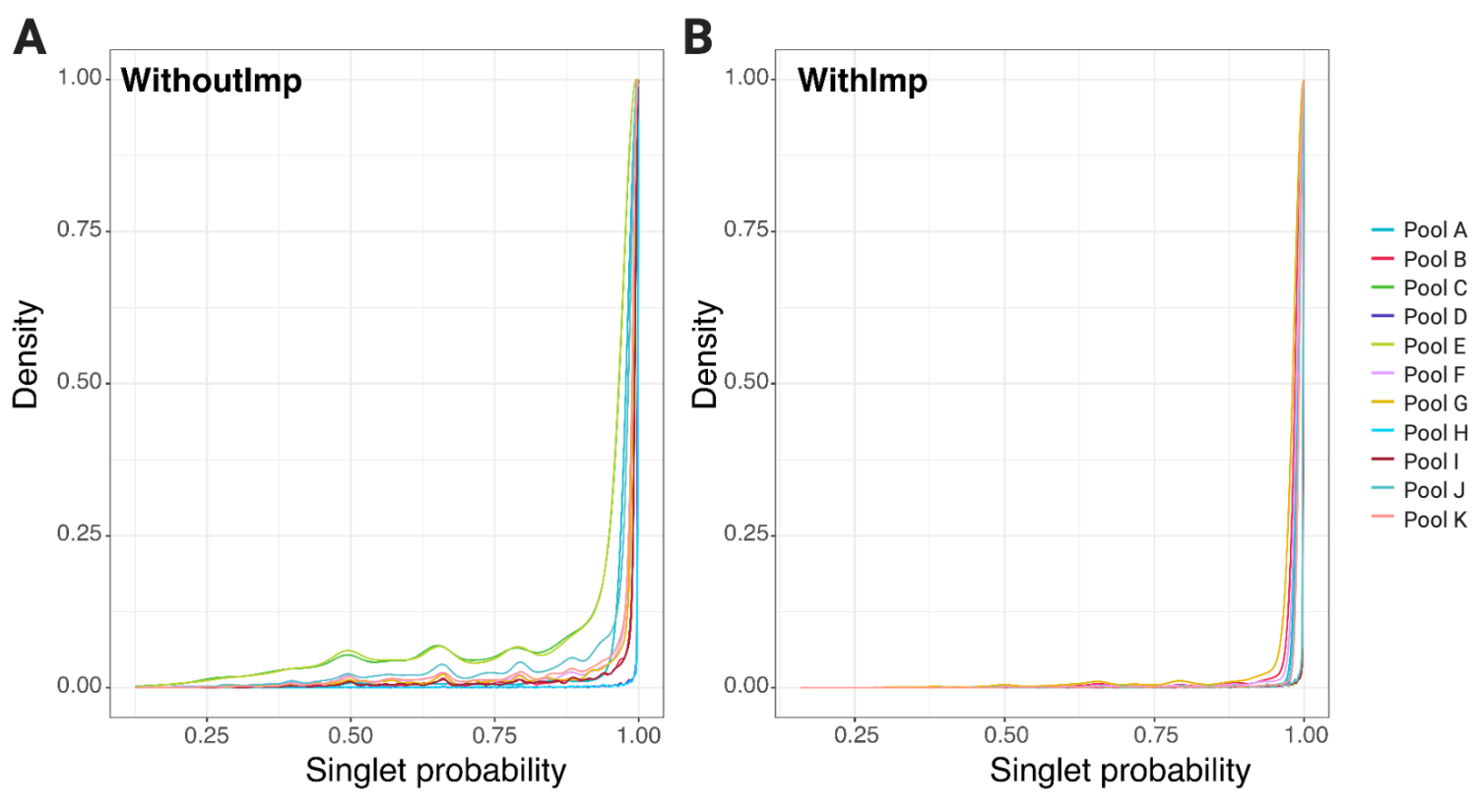


**Figure S17: Distributions of Singlet Probability Without and With Imputed SNP genotypes. A**) Singlet probability distributions were were lower when demuxlet was applied without imputed SNPs (WithoutImp) than **B**) when demuxlet was applied with imputed SNP genotypes (WithImp). SNP: single nucleotide polymorphism.

**
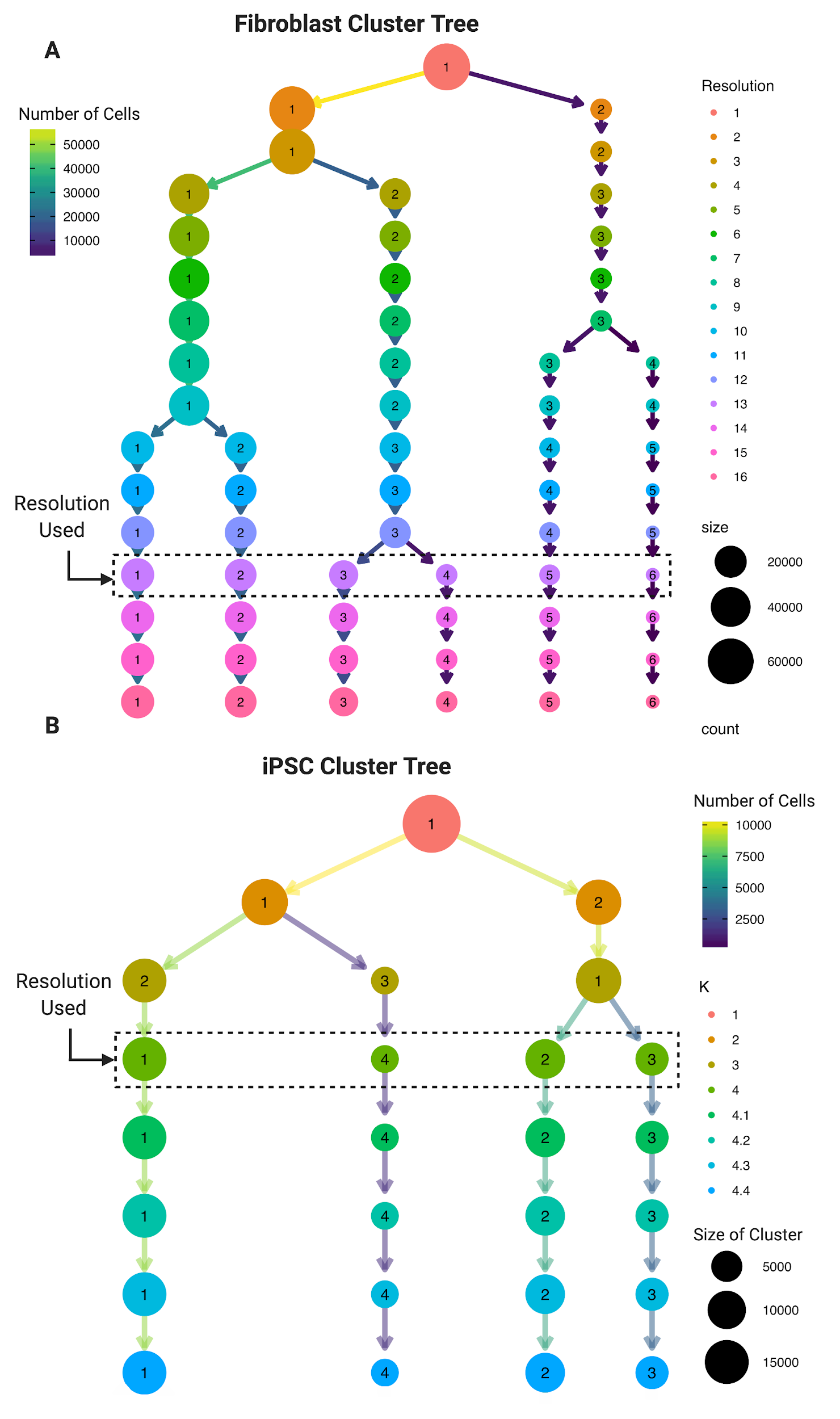
**

**Figure S18: Cluster Tree of fibroblast (A) and iPSC single cell subtypes (B).** Four subtypes of induced pluripotent stem cells (iPSCs) were identified using SCORE clustering. The resolutions used are indicated with dashed boxes on the figures.


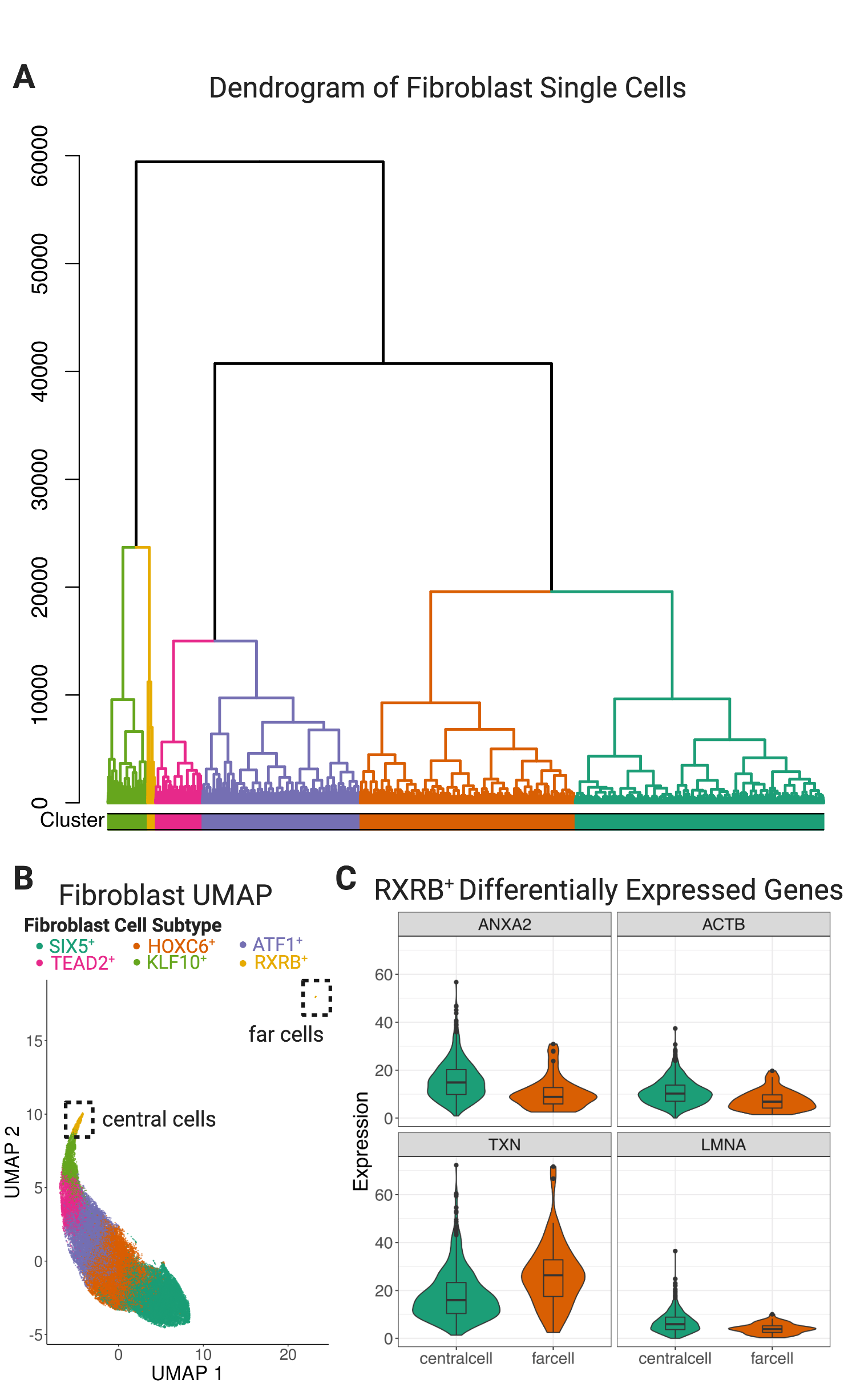


**Figure S19: Investigation of RXRB^+^ cell type. A)** The dendrogram of the cell types demonstrates similar cell structure for the RXRB^+^ cell type compared to the other cell types. **C)** UMAP projection of fibroblast cell types highlighting the two separate groups of RXRB^+^ cell type labeled as “central cells” and “far cells”. **C)** The four significantly differentially expressed genes between the RXRB^+^ “central cells” and “far cells”.


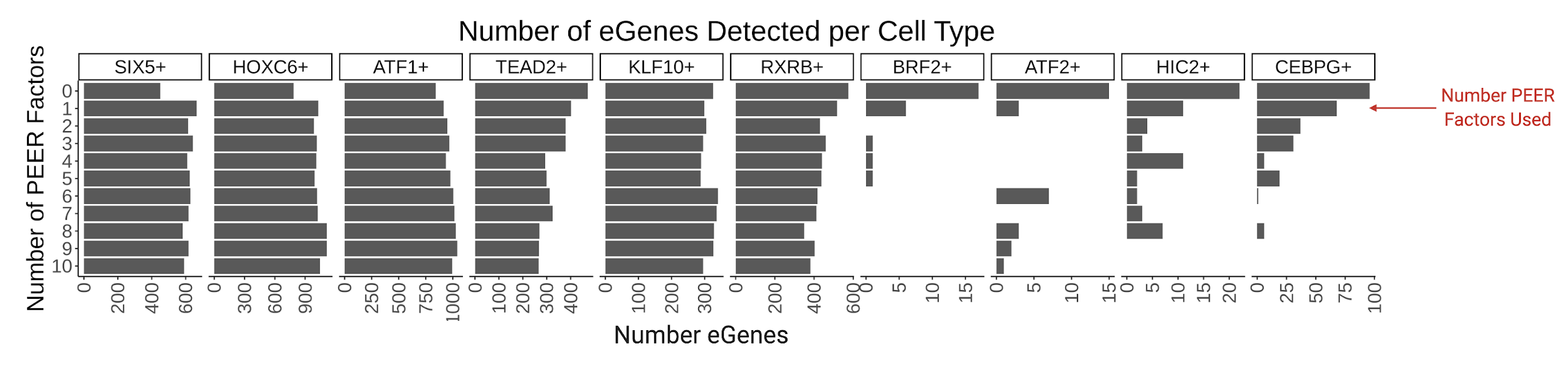


**Figure S20: Number eGenes per Cell Type with PEER factors.** The number of eGenes detected per cell type with different numbers of PEER factors.

**Supplementary Table Legends**

**Table S1: Top 50 transcription factors regulating fibroblast cell types.** The top 50 transcription factors that specifically regulate each of the fibroblast cell types with their relative activity in each cell type.

**Table S2: Top 50 transcription factors regulating iPSC cell types.** The top 50 transcription factors that specifically regulate each of the iPSC cell types with their relative activity in each cell type.

**Table S3: Differentially expressed genes in specific fibroblast types.** The significant differentially expressed genes for each fibroblast type. logFC: log fold change; logCPM: log counts per million; F: F statistic; PValue: P value; FDR: false discovery rate.

**Table S4: Differentially expressed genes in specific iPSC types.** The significant differentially expressed genes for each iPSC type. logFC: log fold change; logCPM: log counts per million; F: F statistic; PValue: P value; FDR: false discovery rate.

**Table S5: Significant fibroblast type eQTLs.** All eSNP-eGene pairs detected as significant in at least one fibroblast type at an FDR threshold of 10%. rsID: reference SNP ID; bp: basepair; MAF: minor allele frequency; FDR: false discovery rate.

**Table S6: Significant iPSC type eQTLs.** All eSNP-eGene pairs detected as significant in at least one iPSC type at an FDR threshold of 10%. rsID: reference SNP ID; bp: basepair; MAF: minor allele frequency; FDR: false discovery rate.

**Table S7: Transcription factor binding motifs that are interrupted by eQTL SNPs.**  eQTL SNPs that were predicted to alter transcription factor binding for the top 50 transcription factors that regulate each of the fibroblast and iPSC types. chr: chromosome; bp: basepair; A1: allele 1; A2: allele 2; rsID: reference SNP ID; TF: transcription factor; FDR: false discovery rate.

**Table S8: eGenes with independent top eQTLs in different cell types.**  eGenes that were detected to have independent eQTLs in different cell types. rsID: reference SNP ID; SE: standard error; P: P-value.

**Table S9: eQTLs that demonstrate SNP-cell type interactions.**  The eSNPs that demonstrate significant eSNP by cell type interactions. rsID: reference SNP ID; bp: basepair; ANCOVA: Analysis of covariance; FDR: false discovery rate;

**Table S10: eQTL models tested.** Results from multiple eQTL models tested for SNPs and genes on chromosome 21. Individual_Average: the average of all cells per individual per cell type. qn: the data was quantile normalized; zt: the data was Z-transformed; 1_PEER_factor: one PEER factor was used as a covariate in the linear model; 10_PEER_factors: ten PEER factors were used as a covariate in the linear model; all_cells: all cells for all individuals were used in the linear model; Individual_Random_Effect: the model was fit with a random effect covariate for the individual who the cell was from.
