# Supplementary material for: Single cell eQTL analysis identifies cell type-specific genetic control of gene expression in fibroblasts and reprogrammed induced pluripotent stem cells
Source: Genome Biol. 2021 Mar 5;22:76. doi: 10.1186/s13059-021-02293-3 (PMC7934233; doi:10.1186/s13059-021-02293-3)
Supplement: Supplementary file 12 — Additional file 12:. Review history. [file 13059_2021_2293_MOESM12_ESM.docx]

Review History

**First round of review**

**Reviewer 1**

**Are you able to assess all statistics in the manuscript, including the appropriateness of statistical tests used?**

Yes, and I have assessed the statistics in my report.

**Comments to author:**

The authors performed scRNA-seq of fibroblasts and iPSCs from human donors, following by mapping expression quantitative trait loci (eQTL). They clustered the cells into several cell types and found that most of the eQTLs are cell type specific. It seems that almost all the eQTLs in fibroblast disappeared in iPSCs. They found that GBP3, which is the only significant eGene shared by 5 from 6 fibroblast cell types, is likely to be mediated by rs541032500 loci. In addition, they suggest that GEMIN5 is an eGene has independent loci in three different cell types. The study is good to present a framework for mapping eQTLs at single cell level and provide a map of cell-type specific eQTLs in fibroblasts and iPSCs. However, this study did not provide enough biological insights to reveal potential function of these eQTLs.

Major issues:

1. The gene expression is very different between fibroblasts and iPSCs, which could result in rare eQTLs shared between them. Although the eQTLs detected in fibroblasts lost in iPSCs, the cell type specific eQTLs may influence the reprogramming efficiency in the early stage. Exploring how the cell type specific eQTLs or eGenes contributing to reprogramming efficiency would be interesting. Similarly, it is likely that the sub-cell types of iPSCs may represent various differentiation-potentials. It is important to know how the cell type specific eQTLs of iPSCs play roles in the self-renew or differentiation of iPSCs.

2. It is known that GBP3 can be induced by IFNγ. It would be interesting that only a subset of cells response to IFNγ. The authors could also detect if other downstream genes of IFNγ have significant eQTLs.

3. Such cell-type specific eQTLs effect might represent the up-stream transcription factors. The author could check if the eQTLs alter the binding of transcription factors or enrich some cell-type specific TF motifs?

Minor issues:

1. Repetitive "that" in line 55 of abstract.

2. The authors should explain what is eGene and eSNP when propose the abbreviation for the first time.

3. The cell types order of Figure 1E should be in line with the clusters order of Figure 1D, just like Figure 1C.

4. The rare cells at the upper right corner of Figure 1B contribute great variance to the UMAP, are they outliers?

**Reviewer 2**

**Are you able to assess all statistics in the manuscript, including the appropriateness of statistical tests used?**

No, I do not feel adequately qualified to assess the statistics.

**Comments to author:**

In this study, Neavin et al. performed an eQTL analysis on ~64,000 fibroblasts from 79 donors and ~20,000 iPSCs from 31 donors. With the recent development of single cell transcriptomics techniques, performing genetic association studies on large cohorts of samples at single cell resolution has become a hot topic and has the potential to result in very interesting findings and novel cell type-specific associations (rather than tissue-specific) between genotype, gene expression and disease. While this manuscript has high-impact potential, there are several issues that need to be addressed.

eQTL detection method needs to be improved:

*	eQTL analysis cannot be performed using a linear model, as in MatrixEQTL, because not all the samples (in the case of this study, cells) are independent. Since each genotype is associated with many cells (>500 cells/individuals), a linear mixed model that takes into account the fact that not all cells are independent from each other needs to be used. If the individuals are unrelated (are they? Please explain in the text), using a method such as lmer in R would be sufficient; otherwise, methods that take the kinship between individuals into account need to be used (limix or lmekin; see reference 25 Cuomo et al. as an example).

*	While the number of tested individuals is low compared to other recent studies, the use of scRNA-seq should improve power to detect eQTLs (testing 64,000 cells should provide more statistical power than testing 79 samples in bulk), but the authors detect a low number of eQTLs for fibroblasts and an extremely low number of eQTLs for iPSCs. More worrisome is the observation that the overlap between eQTLs from different cell types is very low. From GTEx (http://www.ncbi.nlm.nih.gov/pubmed/29022597, see Figure 2b), we expect to observe a bimodal distribution, with many eQTLs shared among all tissues or cell types and others that are tissue- or cell type-specific. Since GTEx identified eQTLs across different tissue types rather than cell types, it should provide a conservative estimate of the number of eQTLs shared between cell types. To determine if the low overlap is due to statistical power, the authors should test the correlation between effect sizes of eQTLs between different cell types.

*	Using PEER factors to remove at least part of the unknown variability between cells should improve the eQTL detection.

*	Removing doublets, rather than force-assigning them to one single individual (line 340-344) should decrease the noise in the eQTL detection.

*	If the cell pooling strategy normalizes gene expression on pools of cells, it might result in flattening the gene expression differences that are due to eQTLs. Rather than selecting the normalization method that best addressed batch effects (which are also addressed by PEER factors), the authors should test which normalization provides the largest number of eQTLs. This could be easily done on a subset of expressed genes.

*	Line 155-160: rather than using such a convoluted method to test if a gene has the same eQTL in two cell types, there are methods that provide this information in a straightforward way (for example colocalization: https://cran.r-project.org/web/packages/coloc/vignettes/vignette.html).

The manuscript would be stronger if the results were discussed in more detail:

*	The authors describe three eGenes in Figure 4, but they provide no interpretation of the interactions that they identify. Are the genes that they describe the ones with strongest interactions?

*	Figure 4A-B show a significant interaction, but the p-value is barely significant at nominal level (p = 0.0147. This p-value should be FDR-corrected for all the tested eGenes. Additionally, the eQTL signal in Figure 4a looks very similar for all cell types, with the two cell types with the most cells (TUBA1Bhi/DCNlo; CD9hi/FTLlo) having the strongest p-values.

*	Figure 4G-I should show p-values.

*	The analyses would be more interesting if the genes described had disease implications.

The scRNA-seq analysis would benefit from a more in-depth discussion:

*	Line 89-93: roughly one third of all the cells map to MSC (mesenchymal stem cells?): do you have an interpretation of this? Figure S1 should be referenced here, rather than in the next sentence. Based on the differential expression analysis, can the authors identify the MSCs as one specific cluster? If not, what is their interpretation?

*	Line 94-95: the gray cluster seem to have a higher fraction of dividing cells. Are there any statistics supporting the statement "Fibroblast and iPSC types contained equal distributions of individual donors, pool batches and cell cycle states"?

*	Line 96-100: how were the key markers chosen? What do the different cells types correspond to in vivo? More description of the different cell types is necessary to understand the results, considering that from the UMAP plot one cannot visually distinguish between clusters.

*	Since there is a good correspondence between cell types and pseudotrajectories, did the authors think about using pseudotrajectory as an additional interaction in the eQTL analysis?

**Authors Response**

**Point-by-point responses to the reviewers’ comments:**

*Specifically, Reviewer #1 highlighted that our manuscript provided an important framework for eQTL mapping from single cell RNA-seq data and provided a map of eQTLs in fibroblast and iPSC cell subtypes. However, they indicated that they would like more biological insight.*

*1) Review #1 inquired about the effect of cell type specific eQTLs on the reprogramming and differentiation efficiency.*

*We agree with the reviewer that this is an interesting and important research question that would be of interest to the broader research community. Unfortunately, the experimental design that we used in this study does not allow us to directly address this question. We have, however, used data from other studies to identify if any of the eQTLs identified in our cell types were in genomic regions that are important for reprogramming or are genes that characterize iPSC reprogramming from fibroblasts. This enabled us to identify a number of loci that have previously been reported as important for iPSC reprogramming that also demonstrate genetic regulation. These results are now included in the revised manuscript on lines 202-216.*

*In addition, we have added additional text to the discussion about future experiments that may be able to address these questions in more detail. As we understand, to do so at the level of cell subtypes would require the measurement of reprogramming efficiency in individual cells, plus an ability to link that measure with the transcriptome of a cell, and thus a cell type. One option that we are aware of to address this would be to use a cell barcoding method (such as CellTag in Biddy et al. 2018 Nature), which each cell is barcoded with a unique DNA tag that is passed onto the daughter cells during reprogramming and differentiation. To address this concept, we have updated the text in the discussion, highlighting both the importance and offering suggestions for new future research on lines 238-242.*

*2) Reviewer #1 was interested that GBP3 was inducible by IFN𝛄 and asked whether other IFN𝛄 inducible genes also had eQTLs.*

*We thank the reviewer for their helpful suggestion. To address this, we have conducted new analysis using the SCENIC regulon method to identify the pathways that were differentially regulated in different fibroblast and iPSC subtypes. This analysis included IFN𝛄 pathway TFs which were not significantly activated in any of the cell subtypes.*

*3) Reviewer #1 pointed out that the cell-type specific eQTLs may be due to cell type specific transcription factor regulation and asked whether the eQTLs altered transcription factor binding motifs.*

*We agree that altered transcription factor binding may be an important mechanism of cell type specific eQTLs. To address this, we tested for eQTLs that were predicted to alter transcription factor binding for the transcription factors that demonstrated cell type specificity from the SCENIC regulon analysis. This enabled us to identify eQTLs for key transcription factors in each cell type that could alter transcription factor binding. These results are included in the main text on lines 118-120 and in Supplementary Table 7 of the revised manuscript.*

*4) Reviewer #1 asked that we remove the repetitive “that” from line 55 of the abstract.*

*We thank the reviewer for identifying this grammatical error and we have now edited the abstract to remove the repetitive “that”.*

*5) Reviewer #1 asked that we explain the terms eGene and eSNP when they are first used in the manuscript.*

*We have now included eGene and eSNP definitions in the manuscript when they first occur. We thank the reviewer for helping us make the manuscript clearer and easier for a reader to understand.*

*6) Reviewer #1 pointed out that the order of the iPSC subtypes was not intuitive for Figure 1E and that suggested that the subtypes should be reordered to follow the same order as their position on the UMAP in Figure 1D.*

*We thank the reviewer for this suggestion and have now reordered the iPSC subtypes in both Figure 1 and all subsequent figures in order to make it more intuitive for the reader.*

*7) Reviewer #1 raised concerns about the cells that were in the upper right hand corner of the fibroblast UMAP from one of the fibroblast cell types (labeld RXR+ in the revised manuscript) and wondered if they might be outliers.*

*We appreciate the reviewers' concerns regarding the cells in the upper right corner of the UMAP. We also had some initial concerns about these cells and conducted a series of analyses to understand whether they are true outliers. Based on this work we have concluded that they are the same cell subtype as other cells presented in the RXR+ subtype. The cells represent 0.04% of the total cells that passed QC, however they remain stabley clustered with the other cells in the RXR+ subtype across all resolutions of the clustering approach (Supplementary Figure 19A of the revised manuscript). In addition, we conducted differential expression analysis between the RXR+ cells in the upper right corner of the UMAP and the rest of the RXR+ cell subtype and found only 4 differentially expressed genes out of 9,796 total genes indicating that there are not extensive differences in their transcriptional profiles (Supplementary Figure 19C of the revised manuscript). This extended information is now included on lines 406-410 of the revised manuscript.*

*We thank Reviewer #1 for their helpful suggestions which we feel have strengthened and clarified our manuscript.*

*Reviewer #2 also found our manuscript to be interesting and to have the possibility to provide important insights about cell type specific eQTLs rather than just tissue-specific eQTLs. However, Reviewer #2 also raised some important concerns.*

*1) Reviewer #2 raised a number of important questions about the model used for eQTL detection and the covariates used in the model.*

*We thank the reviewer for raising this important question. We acknowledge that there is yet an established single method that has been identified as being optimal for single cell eQTL detection. However, to evaluate the effect of different statistical approaches, we tested a variety of methods before selecting the method used for the manuscript - a linear model for expression averaged per individual per cell subtype. During our initial work, we had also considered the use of all the cells with the use of a random effect in a linear mixed model to address the relatedness of each of the cells as the reviewer suggested. However, this method did not identify as many significant eQTLs - none in our test which was run on chromosome 21. To help convey the importance of model choice, we have now included these results as part of the revised manuscript in new Supplementary Table 10.*

*However, it is important to note that the final method we used to test for eQTLs is based on the fact that the individuals used in this study are unrelated (with a mean pairwise Identity by State = 0). For each individual (i) (1-n), for each gene, in each cell-type, we calculate the average expression over m cells measured for that individual. That mean gene expression level is the yi value for individual i, and thus the y vector in the regression model has 1xn dimensions.*

*2) Reviewer #2 asked about the correlation of the betas from eQTLs from one cell subtype in another other cell subtypes.*

*We thank the reviewer for this helpful suggestion and have now included these results in the revised manuscript on lines 159-161 (Supplementary Figure 11).*

*3) The reviewer also suggested the use of PEER to account for any unknown technical noise that could impact the detection of eQTLs.*

*We have now re-perfomed the eQTL analysis with the inclusion of PEER factors to account for unknown technical noise. Because cell lines were pooled for library capture and multiplexed for sequencing, we anticipated that the technical batch variation would be small. As such, we performed 10 rounds of eQTL analysis fitting between one and 10 PEER factors. We found that adding PEER factors enhanced the detection of eQTLs in the fibroblast subtypes but significantly decreased the number of eQTLs detected in the iPSC subtypes. In fibroblasts, fitting 2-10 did not show any major changes in the total number of eQTL detected compared to just one factor (Supplementary Figure 20 in the revised manuscript). Therefore, we elected to use one PEER factor in order to account for the unknown technical noise. These tests and tables have now been added on lines 421-426 of the revised manuscript. The manuscript has been updated with the new numbers of eQTLs, and all secondary analyses have been re-performed resulting in updated figures. We thank the reviewer for providing this suggestion.*

*4) Reviewer #2 also expressed concerns about forcing individual assignments rather than removing doublets.*

*We apologize that we were not clearer in the manuscript but we did remove doublets using demuxlet. We have now clarified the confusing sentence on lines 340-344 of the original manuscript which is now on lines 364-367 of the revised manuscript.*

*5) The reviewer requested that we test a number of eQTL detection methods in order to identify the method that results in the largest number of eQTLs.*

*We agree with the reviewer that the best method for testing for eQTLs in single cell data has not been fully identified. We have now tested a number of different normalization and eQTL detection methods. Those included comparisons of averaged cells per individual per cell type vs all cells, quantile normalizing and z transforming the data or leaving it without transformation and also including various numbers of PEER factors or not using PEER factors. We found that using the average of all cells for an individual for a given cell type followed by quantile normalization and z transformation of the data followed by use of a linear model with one PEER factor was the best model for our data. We have now included these results in the revised manuscript on lines 421-426 and in Supplementary Table 10.*

*6) The reviewer suggested that we consider alternative methods for testing whether two SNPs represent the same locus - specifically, they recommended using the coloc package.*

*We thought carefully about how to test whether two eSNPs for the same eGene may represent the same locus. The coloc package suggested by the reviewer is a great package for comparing effects between different studies and particularly for associations that are on different scales. However, in our case, all associations between SNPs and gene expressions as well as data processing for the data were carried out at the same time. Therefore, we implemented the same method used for conditional eQTL detection - regress out one SNP, thereby removing the variation in gene expression accounted for by that SNP and then test for significant association with other SNPs. This is an effective method to identify if two or more SNPs may be effectively tagging the same locus. These results are presented in Figure 3 and on lines 175-180 of the revised manuscript.*

*7) Reviewer #2 requested that the results be discussed in more detail - specifically the results in Figure 4 of the manuscript.*

*We appreciate the reviewers interest in having more details about our results, how they relate to current knowledge and their biological relevance. We have included just two examples in Figure 4 in order to be able to focus on two examples in more detail and have included more details about those interesting loci such as their currently understood functions and how genetic variation might contribute to their functions during reprogramming and differentiation. We have included these details on lines 184-191 and 218-223.*

*8) The reviewer requested that Figure 4 show p-values instead of * to indicate p-values.*

*We have now changed the p-value nomenclature in Figure 4 to include FDR corrected p-values instead of the use of asterisks.*

*9) The reviewer asked whether the interaction effects described and the p-values shown were corrected for multiple comparisons.*

*We have corrected the interaction p-values using FDR Benjamini–Hochberg procedure. We thank the reviewer for pointing out that this was not clear and have included additional text to clarify this in the revised text on lines 198-199.*

*10) Reviewer #2 was intrigued that ~⅓ of our fibroblast single cells mapped to mesenchymal stem cells (MSCs) and was curious whether this corresponded to a specific subtype of fibroblasts identified in our study.*

*We appreciate the interest of the reviewer in this single cell classification and we have altered the text and figures to address these comments. It has been well described that MSCs and fibroblasts are extremely similar in their phenotypes, appearance and transcriptional profiles. Therefore, it is not surprising that many fibroblast cells map to MSCs. To help demonstrate this, we have also now included a PCA plot demonstrating the similarity of the fibroblast and MSC transcriptional profiles (Supplementary Figure 5 of the revised manuscript). In addition, we have also included the proportion of each mapped category that were from each fibroblast subtype which demonstrated that relatively equal proportions of each cell subtype were classified in both fibroblasts and MSCs. In conclusion, no fibroblast subtypes were preferentially mapped to MSCs. This is included on lines 101-107 of the revised manuscript.*

*11) The reviewer was concerned that one of the iPSC cell types had more cycling cells than the other three and asked for statistics supporting our claims that there were similar numbers of different cell cycles across the 4 iPSC subtypes*

*The reviewer raises a very valid concern regarding the cycling cells. Even though the HIC2+ iPSC subtype appears to have more cycling cells on the UMAP, they do not contain statistically more cycling cells (tested with a Kruskall-Wallis test). We have included this information in Supplementary Figure 2 and on lines 94-96 in the revised manuscript.*

*12) Reviewer #2 also raised a question regarding the method of markers chosen for cell subtype naming.*

*We appreciate this question from the reviewer. After we ran regulon detection with SCENIC based on some of the questions posed by Reviewer #1, we felt that the cell types would be more accurately named after the regulons that were specifically activated in each cell type. Therefore, we have renamed them based on those regulons (Figure 1C, E) and included updated text to describe this in the revised manuscript on lines 96-97.*

*13) The reviewer asked whether we had considered including pseudotrajectory analysis as an interaction term for eQTL detection.*

*We found this suggestion to be very helpful. However, this would only be possible if the eQTLs were detected using all cells instead of the average of the cells per individual per cell type. Since we identified that averaging the cells per individual per cell type was the best method to identify the most eQTLs, including the pseudotrajectory analysis as an interaction term was not a possibility. However, we used another approach to answer this question by testing whether neighboring cell types shared a larger percentage of significant eGenes than more distant cell types. We found that neighboring clusters did indeed share more significant eGenes than distant cell types (Figure 2B-C and lines 114-117 of the revised manuscript). This indicates that, as the reviewer suggests, neighboring cell types are more likely to share eQTLs than distant cell types.*

*We thank Reviewer #2 for their comments, questions and suggestions.*

*We found the comments from both reviewers to be insightful and feel that they have resulted in a clearer and more effective revised manuscript. We hope that the reviewers find that our additional analyses have addressed their main concerns and we hope that you will consider publishing our revised manuscript in Genome Biology.*

**Second round of review**

**Reviewer 1**

The authors have in general addressed my concerns. The manuscript is suitable publication in Genome Biology.

**Reviewer 2**

After looking at the edited manuscript, I still have a few major concerns:

1.	In my second comment, I raised concerns about “More worrisome is the observation that the overlap between eQTLs from different cell types is very low”. The authors still do not provide any explanation on why eQTLs in different iPSC cell types, which are in general highly homogeneous, do not overlap. Most likely, this is a matter of power, because all the iPSC cell types are highly similar to each other and this needs to be mentioned. However, the authors may have other explanations and/or interesting biological insights and they should describe them thoroughly at least in the Discussion. In the discussion they may consider contrasting to the fact that GTEx shows significant overlap between eQTLs from different tissues, which are much more heterogeneous compared with iPSC cell types.

2.	Further comment regarding point 1 above: “Reviewer #2 asked about the correlation of the betas from eQTLs from one cell subtype in another other cell subtypes.”: looking at Figure S11, it is very interesting to observe that in iPSCs there is absolutely no correlation between the effect sizes of eQTLs in any pair of cell types. If the corresponding eGenes are expressed in both cell types, this would suggest that the eQTL differences between cell types are not due to power, but they reflect biological differences. The Discussion should describe this and its potential implications in detail.

3.	The methods lack most of the details on the new analyses done in response to reviewers’ comments (see below)

4.	Data is not available anywhere and no links or IDs to databases such as GEO or dbGaP or ENA/EGA are provided.

Details on the methods

a.	“However, in our case, all associations between SNPs and gene expressions as well as data processing for the data were carried out at the same time. Therefore, we implemented the same method used for conditional eQTL detection - regress out one SNP, thereby removing the variation in gene expression accounted for by that SNP and then test for significant association with other SNPs. This is an effective method to identify if two or more SNPs may be effectively tagging the same locus. These results are presented in Figure 3 and on lines 175-180 of the revised manuscript.”

We disagree with the authors on this point. With colocalization, one should be able to calculate the probability that two eQTL signals for the same gene (one from cell type 1 and the other from cell type 2) are the same. If the authors performed one single eQTL analysis, how can they have cell type-specific eQTLs? The methods do not explain this.

b.	The authors claim in their response that eQTL analysis was performed on the mean expression across all cells from the same individual. In this case, how was the expression determined? This is not described in the Methods.

c.	The authors describe in their response that they tried multiple ways to determine eQTLs (Table S10), but they do not describe how they performed these tests. Different types of normalizations were performed and PEER factors were calculated (how? Using the expression of how many genes?). The authors should describe this in the Methods in detail.

d.	The authors correctly addressed my comment number 9. “The reviewer asked whether the interaction effects described and the p-values shown were corrected for multiple comparisons” They describe that they used Benjamini-Hochgberg’s correction but this information should also be provided in the Methods section.

**Authors Response**

**Point-by-point responses to the reviewers’ comments:**

Below is a reply to the reviewer’s comments.

1) Reviewer #2 requested that we provide more context for our findings vs bulk RNA-seq finding from GTEx and explain that the signals may be truly biological cell type-specific eQTLs.

*We appreciate the reviewer’s interest in the relationship of single cell RNA-seq eQTL detection vs bulk RNA-seq eQTL detection. We have edited the manuscript to include a paragraph (lines 237-248) in the Discussion that provides context for the reader. One point that we feel is important to make is the limitations in power due to sample size for adequate elucidating the differences between bulk and single cell RNA-seq eQTL detection.*

2) The reviewer pointed out that we had failed to include many details of the new analyses in the Methods section.

*We thank the reviewer flagging this with us. We apologize for failing to include important details for the new analyses in the Methods section in our last revision. We have now updated the methods with these details (lines 429-443 and 461-490).*

3) The reviewer indicated that we had not yet provided the data publicly.

*The scRNA-seq data for all 79 fibroblast cell lines and 31 iPSC cell lines have been deposited to ArrayExpress and is publicly available under the accession number E-MTAB-10060 (lines 612-613).*

4) The reviewer indicated that we were not clear about the processing for the expression values that were used in the model to detect eQTLs.

*We apologize that we were not clear about the method used to obtain the expression values used for eQTL detection. We generated a single expression value (yi) per gene per individual per cell type by averaging the expression levels of the N cells from that individual for each given cell type. We have now clarified this in the Methods section (lines 486-490).*

5) Reviewer #2 requested that we provide additional details about the various eQTL detection methods performed.

*We thank the reviewer for their suggestion and have now included additional details for the eQTL models that were tested in the Methods section (lines 461-484).*

6) The reviewer requested that we explicitly state that the Benjamini-Hochberg method was used for multiple testing correction.

*We have now included this information in the Methods section (line 490).*
